# GADD45β inhibits RIPK3-mediated NF-κB activation by interfering with NEMO-RIPK1-RIPK3 interactions

**DOI:** 10.1038/s41420-025-02894-y

**Published:** 2025-12-07

**Authors:** Carmela Casale, Alete Colella, Miriam Cruoglio, Serena Mirra, Emanuela Iaccarino, Maria Brigida Lioi, Francesca Fusco, Annamaria Sandomenico, Antonio Leonardi, Francesca Zazzeroni, Alessandra Pescatore

**Affiliations:** 1 Institute of Genetics and Biophysics ‘Adriano Buzzati-Traverso’ (CNR), Naples, Italy; 2 Department of Biology and Evolution of Marine Organisms, Stazione Zoologica Anton Dohrn, Naples, Italy; 3 Institute of Biostructures and Bioimaging, CNR, Naples, Italy; 4 Department of Science, University of Basilicata, Potenza, Italy; 5 Dipartimento di Medicina Molecolare e Biotecnologie Mediche, University of Naples Federico II, Naples, Italy; 6 Department of Biotechnological and Applied Clinical Sciences (DISCAB), University of L’Aquila, L’Aquila, Italy

**Keywords:** Necroptosis, Cell death and immune response

## Abstract

Necroptosis is a highly inflammatory form of regulated cell death driven by Receptor-Interacting Protein Kinase 3 (RIPK3), which plays a crucial role in immune responses, inflammatory diseases, and tumor microenvironment modulation. Beyond driving cell death via MLKL phosphorylation, RIPK3 also activates NF-κB signaling, promoting cytokine production and immunogenic responses. However, the regulatory mechanisms governing RIPK3-dependent NF-κB activation remain largely unclear. Here, we identify Growth Arrest and DNA Damage-inducible β (GADD45β) as a novel regulator of RIPK3 activities. We show that GADD45β directly binds RIPK3 in a RHIM-independent manner, interfering with NEMO-RIPK1-RIPK3 complex formation and limiting RIPK3-mediated NF-κB activation. Furthermore, inducible expression of GADD45β selectively suppresses RIPK3-induced proinflammatory signaling without promoting caspase-dependent apoptosis and markedly reduces CXCL8 (IL-8) production during necroptotic stimulation. GADD45β also improves long-term cellular survival under sustained inflammatory stress. Our findings reveal GADD45β as a critical modulator of RIPK3-driven immune responses and suggest a potential therapeutic strategy for fine-tuning immunogenic cell death.

## Introduction

Necroptosis is a highly inflammatory form of regulated cell death (RCD), characterized by cellular swelling and membrane rupture, leading to the release of damage-associated molecular patterns (DAMPs) that amplify immune responses [1–4]. It is triggered by TNFR, IFNαR, TLR ligands, viral infections, and genotoxic stress, and often occurs under apoptosis-deficient conditions. In such settings, necroptosis provides a critical defense against intracellular pathogens and helps shape immune responses during infection, tissue injury, and cancer [5–11]. Necroptosis depends on the activation of RIPK3 and the phosphorylation of its downstream effector, the mixed-lineage kinase domain-like protein (MLKL) [12].

Mechanistically, necroptosis is induced under caspase-deficient conditions, such as through Caspase-8 inhibition by viral proteins (e.g., vICA from MCMV) or in the presence of pharmacological pan-caspase inhibitors (e.g., zVAD-fmk). RIPK3 activation depends on its RHIM domain, which enables the formation of functional amyloid structures essential for its activation [13, 14]. This conserved domain regulates RIPK1-RIPK3 interactions and modulates RIPK3 activation through additional RHIM-containing adapters [15, 16]. RIPK1 preferentially binds to RIPK3, initiating hetero-amyloid formation, which then acts as a nucleation core for RIPK3 homo-amyloid assembly, amplifying necroptotic signaling with minimal RIPK1 input [17, 18]. Post-translational modifications of RIPK1, RIPK3, and MLKL dynamically regulate necroptotic signaling by influencing protein conformation and activity [19]. In addition to executing necroptosis via MLKL, RIPK3 also drives pro-inflammatory and pro-survival programs through NF-κB activation [20–22]. Notably, in contexts of low expression RIPK3 activation, cells can sustain NF-κB-driven inflammatory responses without immediate lysis, contributing to chronic inflammation and tumor progression. This state has been referred to as “sublethal” necroptosis [23, 24]. The critical role of RHIM-mediated interactions in necroptosis is underscored by the fact that diverse pathogens have evolved mechanisms to disrupt this pathway, including viral RHIM mimics and bacterial proteases that target RHIM-containing proteins to inhibit RIPK3 activation [25–27]. While the necroptotic role of RIPK3 is well-characterized, the regulatory mechanisms that fine-tune its inflammatory functions remain poorly defined.

Growth Arrest and DNA Damage-inducible beta (GADD45β) is a stress-responsive, NF-κB-inducible gene known to modulate inflammation and tumor progression by inhibiting MKK7 to block JNK signaling and influence macrophage polarization and T-cell recruitment [28–31]. Recent evidence shows that GADD45β can form amyloid-like aggregates under physiological conditions [32], raising the possibility of physical interactions with other amyloidogenic proteins. However, whether GADD45β directly regulates RIPK3-driven processes has remained unexplored.

Here, we investigate how GADD45β modulates RIPK3-driven necroptotic signaling. We demonstrate that GADD45β directly binds to RIPK3 and interferes with RHIM-mediated complex formation, thereby attenuating RIPK3-dependent NF-κB canonical activation. This interaction dampens downstream proinflammatory cytokine production, including CXCL8, without affecting RIPK3 expression or localization. Our findings reveal GADD45β as a context-specific regulator of necroptotic inflammation, acting through selective inhibition of RIPK3-associated intracellular signaling mechanisms.

## RESULTS

### GADD45β interferes with RIPK3-RIPK1 complex formation and RIPK3 self-association via direct binding

**Fig. 1:**
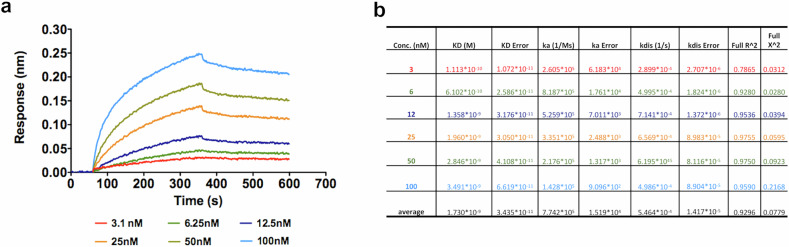
Biolayer Interferometry (BLI)-Based Biophysical Assays of GADD45β binding to RIPK3. **a** BLI sensorgrams showing the binding curves of recombinant human GADD45β (rhGADD45β) at indicated concentrations to recombinant human RIPK3 (rhRIPK3). **b** Graphical summary of kinetic parameters and apparent affinity constants (KD) derived from BLI experiments, analysed using Octet Analysis Studio 12.2.

GADD45β is known to regulate stress and inflammatory signaling, yet its potential role in necroptosis remains unclear. Given the central function of RIPK3 in necroptotic signaling and its regulation through RHIM-mediated interactions, we hypothesized that GADD45β may modulate RIPK3 activity through direct binding. To evaluate this, we performed biolayer interferometry (BLI) kinetic binding assays to assess the interaction between recombinant human GADD45β and RIPK3 (Fig. 1a). As shown, rhGADD45β bound to rhRIPK3 in a dose-dependent manner in the range 3.1–100 nM, with a calculated affinity constant (KD) of 1.73 ± 0.03 × 10^–9 ^M (Fig. 1b).

**Fig. 2:**
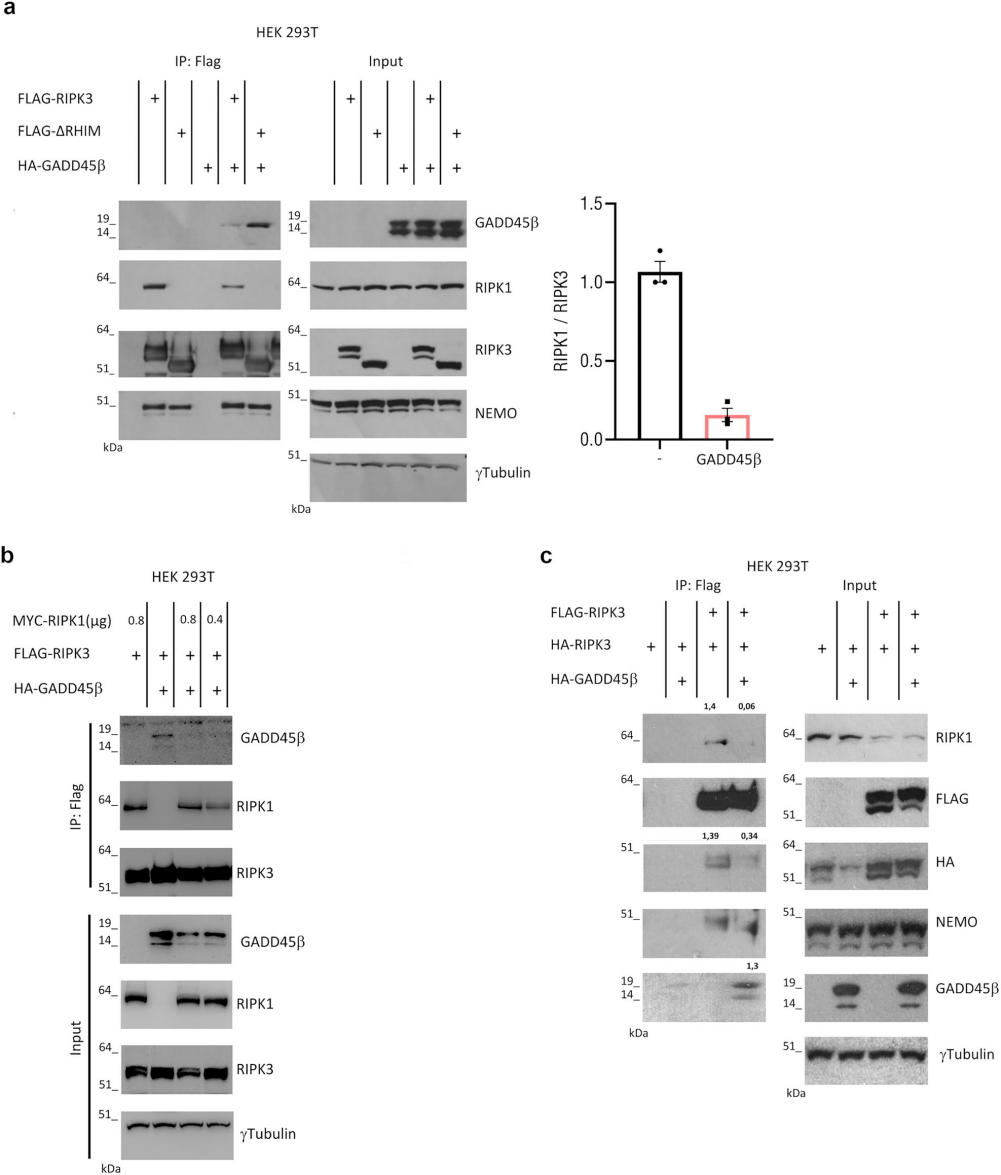
Interaction between RIPK3 and GADD45β in HEK293T Cells. **a** Immunoprecipitation (IP) of Flag-tagged RIPK3 wild-type or ΔRHIM mutant expressed in HEK293T cells with or without HA-tagged GADD45β. IP performed using anti-Flag antibody. Immunoblot shows indicated proteins. Quantification of RIPK1/Flag-RIPK3 ratio (black: without GADD45β; pink: with GADD45β). The means of three independent experiments (*n* = 3) are displayed as bars. **b** HEK293T cells were transfected with different concentrations of MYC-RIPK1 plasmid in presence and absence of Flag-RIPK3 and GADD45β-HA plasmids. Lysates were subjected to anti-Flag immunoprecipitation; immunoblot analysis was performed to identify the indicated proteins. Representative experiment of two is shown. **c** HEK293T cells were transfected with the indicated plasmids, lysates were subjected to anti-Flag immunoprecipitation, immunoblot analysis was performed to identify the indicated proteins. The Protein ratios relative to Flag-RIPK3 indicated in the blot. Representative experiment of three is shown.

Next, we sought to determine whether this interaction occurs in a cellular context. We first analysed the interaction of GADD45β with either full-length RIPK3 or a truncated form lacking the RIP homotypic interaction motif (ΔRHIM). The RHIM domain is required for the recruitment of RIPK3 to RIPK1, forming stable fibrils essential for necroptotic signal transduction [17, 33]. We report that GADD45β binds to RIPK3 independently of the RHIM domain and significantly inhibits the interaction between RIPK1 and RIPK3, thereby reducing their association (Fig. 2a). In previous work, we showed that NEMO functions as a scaffold that restricts RIPK3 kinase activity [34]. Consistent with this finding, GADD45β did not impair the interaction between NEMO and either full-length RIPK3 or the RHIM-deleted mutant, suggesting that GADD45β selectively interferes with RHIM-mediated complex formation without affecting NEMO recruitment (Fig. 2a). Remarkably, the interaction between GADD45β and the RHIM-deleted RIPK3 mutant (ΔRHIM), which is unable to bind RIPK1, was stronger than with the full-length RIPK3. This supports a model in which GADD45β and RIPK1 may compete for overlapping or mutually exclusive binding surfaces on RIPK3. Consistent with this, overexpression of RIPK1 restored the RIPK1-RIPK3 interaction even in the presence of GADD45β (Fig. 2b), indicating stoichiometric competition between RIPK1 and GADD45β. Since RIPK1 and RIPK3 interact via their RHIM domains, and RHIM-mediated complex formation is a prerequisite for downstream RIPK3 kinase activation [2], we reasoned that GADD45β might additionally disrupt RIPK3 self-association.

To test this, we performed co-immunoprecipitation assays using Flag-tagged RIPK3 and HA-tagged RIPK3 in the presence or absence of GADD45β. The results showed that GADD45β reduced RIPK3-RIPK3 interaction (Fig. 2c). Together, these data demonstrate that GADD45β disrupts both RIPK1-RIPK3 complex formation and RIPK3 self-association through binding, identifying GADD45β as a novel negative regulator of RHIM-dependent signaling.

### GADD45β inhibits RIPK3-mediated canonical NF-κB activation in a dose-dependent manner

**Fig. 3:**
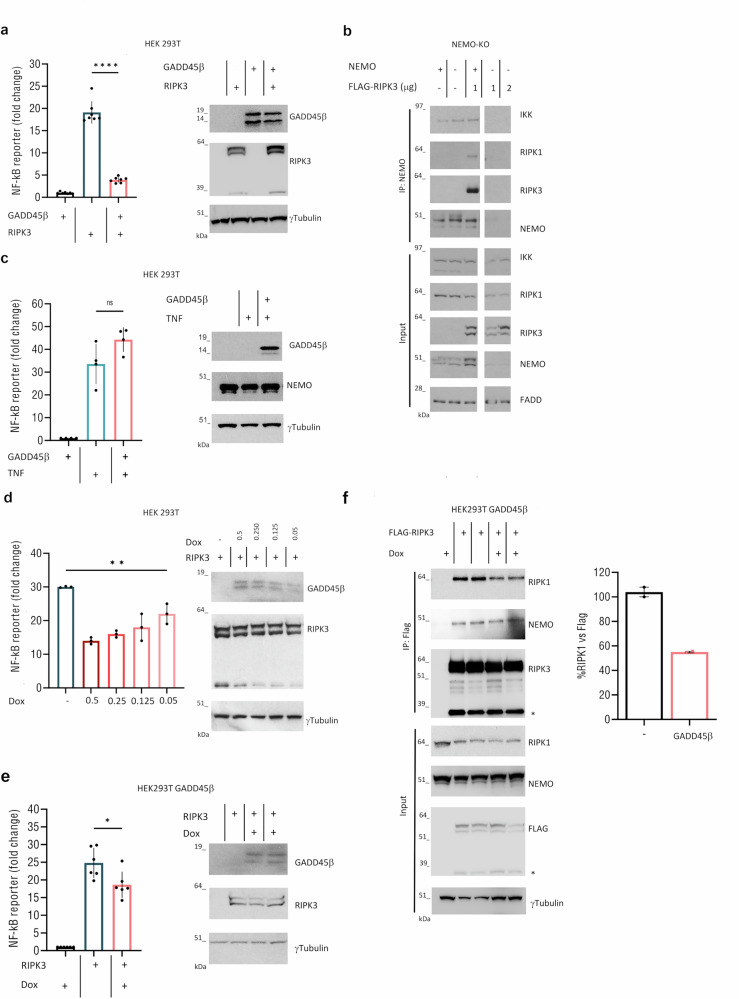
GADD45β inhibits RIPK3-mediated NF-κB activation via a dose-dependent mechanism. **a** HEK293T cells were transfected with an NF-κB luciferase reporter plasmid along with expression vectors for RIPK3, GADD45β, or an empty vector (EV). Luciferase activity was measured 24 h (h) post-transfection, and fold induction was calculated relative to EV control Data represent individual values with mean ± SEM from seven independent experiments (*n* = 7; *p* < 0.0001). **b** Flag-RIPK3 was expressed in HEK293 NEMO-null (KO) cells, with or without NEMO reconstitution. Interaction between RIPK3 and NEMO Interaction analysed by anti-NEMO IP and immunoblotting. **c** HEK293T cells were transfected with GADD45β or EV stimulated with TNFα (10 ng/mL) for 4 h. NF-κB activity measured by the luciferase reporter assay. Data represent mean ± SEM from four independent experiments (*n* = 4; *p* = 0.084). **d** HEK293T cells expressing RIPK3 and a doxycycline (Dox)-inducible pLVX-TetOne-GADD45β plasmid were treated with the indicated concentrations of Dox. Luciferase activity was measured as above. The means of three independent experiments (*n* = 3) are displayed as bars (*p* = 0.01). **e** Dox-inducible GADD45β HEK293T cells transfected as in (**a**), treated ± Dox (1 μg/mL). NF-κB activation shown as mean ± SEM from six independent experiments (*n* = 6, *p* = 0.023), and **f** Co-IP of RIPK3 ± Dox-induced GADD45β expression. RIPK1/Flag-RIPK3 ratios are expressed as percentages relative to the control condition without GADD45β (set to 100%). The means of two independent experiments are displayed as bars. Black: without GADD45β; pink: with GADD45β. Unless otherwise specified, data are analysed using unpaired, two-tailed Student’s *t* tests. Significance: *p < 0.05; **p < 0.01; ***p < 0.001; ****p < 0.0001; ns, not significant (*p* > 0.05).

Previous studies RIPK3 showed that RIPK1-RIPK3 interaction is essential for NF-κB activation in both humans and mice [20, 35–37]. Therefore, we investigated whether GADD45β modulates RIPK3-dependent NF-κB activation. Using a luciferase reporter system, we observed that co-expression of GADD45β resulted in an ~80% reduction in RIPK3-induced NF-κB activity (Fig. 3a). RIPK3’s ability to activate NF-κB depends on its RHIM domain. Although the kinase-dead RIPK3 mutant (D160N) is incapable of triggering necroptosis, it retains the ability to activate NF-κB via RHIM-mediated mechanisms, a process that was also suppressed by GADD45β (Supplementary Fig. S1a). Since the precise mechanism by which RIPK3 oligomerization leads to RIPK1-dependent NF-κB signaling remains unclear, we assessed whether this activation involves the IKK complex. Overexpression of RIPK3 in HEK 293 NEMO-null cells (NEMO-KO) cells failed to induce NF-κB activity, while robust activation was observed in NEMO-proficient cells, confirming that RIPK3 signals through the canonical NF-κB pathway (Supplementary Fig. S1b). Mass spectrometry data previously identified NEMO in RIPK3 immunoprecipitates [38], supporting its involvement in RIPK3-associated complexes. Accordingly, we showed that RIPK3 forms a complex with NEMO, RIPK1, and IKK (Fig. 3b), and hypothesized that GADD45β may interfere with the formation or stability of this multiprotein complex. To test this, we immunoprecipitated Flag-tagged NEMO and consistently observed co-immunoprecipitation of RIPK3 and IKK. However, in the presence of GADD45β, the amount of RIPK3 bound to NEMO was markedly reduced (Supplementary Fig. S2a). This imply that GADD45β does not prevent the initial binding of RIPK3 to NEMO but impairs the assembly or stabilization of RIPK3-NEMO-IKK complexes, thereby modulating downstream NF-κB activation. Consistent with this hypothesis, GADD45β did not disrupt RIPK1-RIPK3 interaction in NEMO-KO cells (Supplementary Fig. S2b). Although RIPK3 was detected in both NEMO and GADD45β immunoprecipitated, reciprocal co-IP assays showed no interaction between NEMO and GADD45β, reinforcing that NEMO is dispensable for GADD45β-RIPK3 complex formation (Supplementary Fig. S2a, c). Furthermore, since TNF-induced NF-κB activation relies predominantly on RIPK1, we tested whether GADD45β influences this axis. The results showed that GADD45β selectively targets RIPK3-driven, but not RIPK1-mediated, NF-κB activation (Fig. 3c). To assess the specificity and dose-dependency of this effect, we employed a doxycycline-inducible expression system where incremental GADD45β expression proportionally reduced RIPK3-mediated NF-κB activation (Fig. 3d). This finding was further validated in puromycin-resistant HEK293T cells stably transduced with a doxycycline-inducible GADD45β construct, in which even low levels of GADD45β expression reduced RIPK3-RIPK1 interaction (Fig. 3e, f), supporting a dose-dependent inhibitory role for GADD45β in RIPK3-driven canonical NF-κB signaling.

### GADD45β interferes with p65 nuclear translocation and cell fate during necroptosis in colon cancer cells

**Fig. 4:**
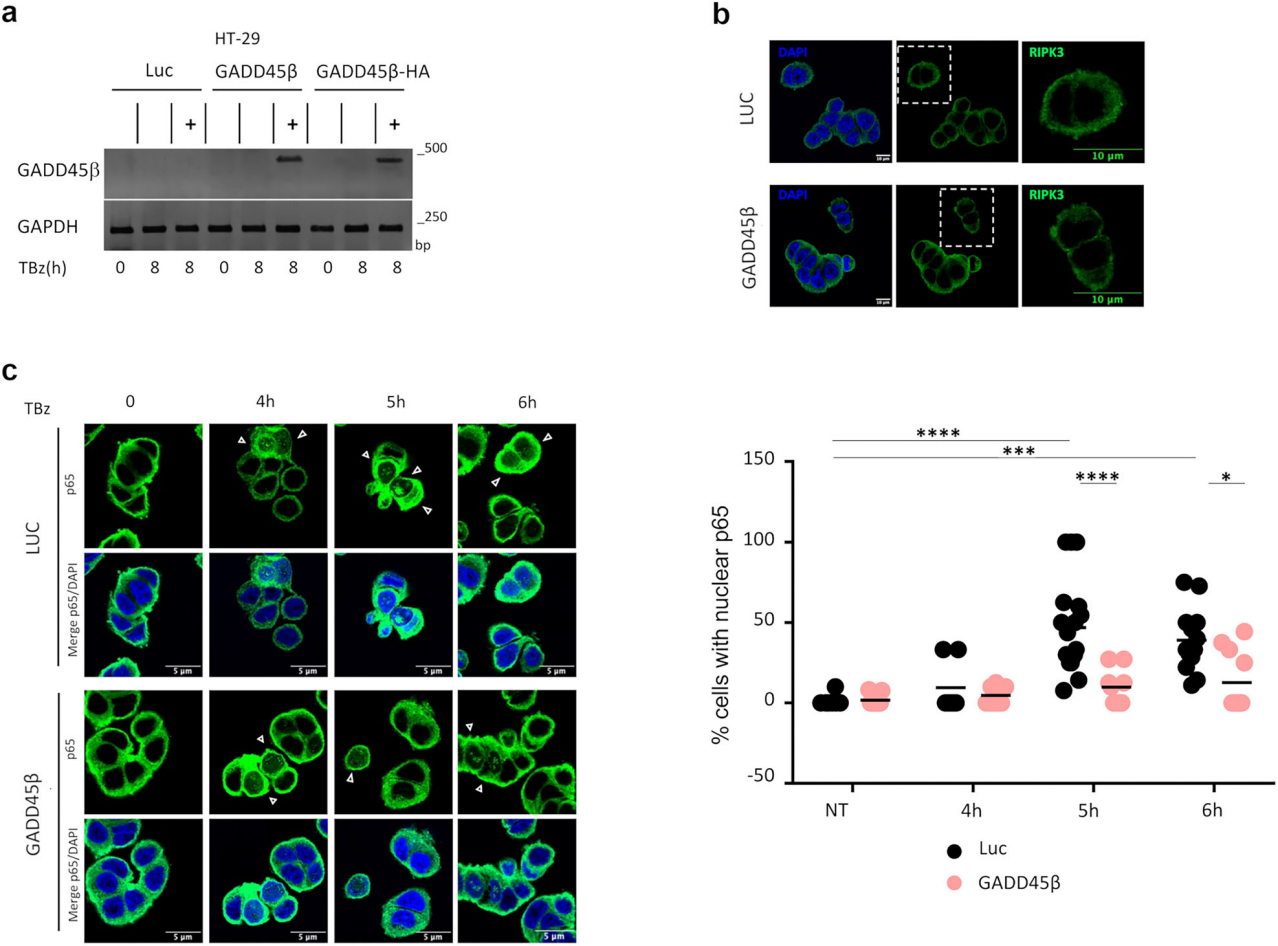
GADD45β interferes with p65 nuclear translocation. **a** HT-29 cells stably transduced with a doxycycline-inducible pLVX-TetOne-Luciferase (Luc), pLVX-TetOne-GADD45β (GADD45β), or pLVX-TetOne-GADD45β-HA (GADD45β-HA) treated or not with 1 μg/mL Dox for 24 h. GADD45β mRNA levels assessed by reverse transcription-polymerase chain reaction (RT-PCR). **b** Representative confocal microscopy images of HT-29 Luc and GADD45β cells stained for RIPK3 (Alexa Fluor 488, green) and nuclei (DAPI, blue). Scale bar: 10 μm. **c** In left, confocal images of HT29 Luc and GADD45β cells treated with TBz (TNF, BV6, zVAD) for the indicated time periods in the presence of Dox (1 μg/mL) and stained for p65 (Alexa Fluor 488, green) and nuclei (DAPI, blue). White arrows indicate cell with nuclear p65. Scale bar: 5 μm. In right, quantification of the percentage of cells with nuclear p65, based on 7–18 fields per condition (68–152 cells per condition). Data mean ± SEM. Statistical significance: *p ≤ 0.05; ***p ≤ 0.001; **p ≤ 0.0001 (Two-way ANOVA with Tukey’s post hoc test).

**Fig. 5:**
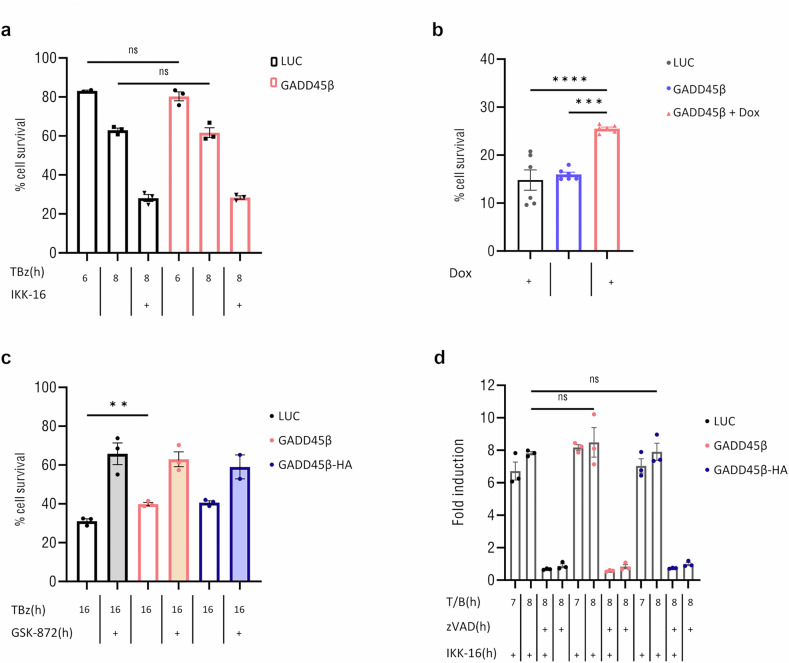
GADD45β promotes long-term survival under necroptotic conditions via RIPK3 inhibition. **a** HT-29 Luc and GADD45β-inducible cells were stimulated with TBz for 6 and 8 h and with TBz/IKK-16 for 8 h, all in the presence of 1 μg/mL Dox. Cell viability was measured using the CellTiter-Glo assay and is expressed as a percentage relative to the untreated control. Data represent mean ± SEM from three independent experiments (*n* = 3). **b** Cells as in (**a**) were stimulated with TBz for 18 h in the presence or absence of 1 μg/mL Dox. Cell viability was measured as in (**a**) and is expressed relative to the untreated (no TBz) control. Data represent mean ± SEM from six independent experiments (*n* = 6). One-way ANOVA with Tukey’s multiple comparisons test; ****p < 0.0001; ***p = 0.0002. **c** Luc, GADD45β and GADD45β-HA cells were stimulated with the indicated inhibitors and cell viability was measured following GSK-872 treatment, all in the presence of 1 μg/mL Dox. **d** Same cells as in (**c**) were stimulated with indicated inhibitors, and caspase-3/7 activity is expressed as fold induction relative to untreated controls; **Student’s *t* test; *p* < 0.001.

Although GADD45β is a known NF-κB target gene, our findings suggest that it functions as a negative regulator of RIPK3 by inhibiting RIPK3-mediated NF-κB activation. In HT-29 colon cancer cells undergoing TNFα-induced necroptosis in the presence of the second mitochondria-derived activator of caspases mimetics and the caspase-8 inhibitor zVAD, TNF stimulation triggers a biphasic NF-κB activation, characterized by sustained nuclear accumulation of p65 that promotes proinflammatory cytokine transcription. This process depends on both the scaffolding function of RIPK1 and the RIPK3-MLKL axis. This response is characterized by two distinct waves of p65 nuclear translocation: an early, transient phase occurring within 15–30 min after stimulation, and a delayed, sustained phase between 4 and 8 h, which temporally coincides with cytokine gene expression and necroptotic execution [39]. To investigate the role of GADD45β in this process, we generated HT-29 cells with doxycycline-inducible expression of untagged GADD45β or a C-terminally HA-tagged version (GADD45β-HA) (Fig. 4a). In cells expressing GADD45β, RIPK3 expression and subcellular localization remained unchanged (Fig. 4b). To assess whether GADD45β interferes with either phase of NF-κB activation, we performed time-course experiments examining p65 localization dynamics. At early timepoints (0, 0.25, and 0.5 h) following TBz (TNF, BV6, zVAD), no significant differences in nuclear enrichment of p65 were detected by immunofluorescence (Supplementary Fig. S3a, b). In contrast, GADD45β markedly reduced p65 nuclear translocation between 5 and 6 h after TBz treatment, as demonstrated by immunofluorescence and confirmed by nuclear/cytoplasmic fractionation and immunoblotting for p65 (Fig. 4c and Supplementary Fig. S3c). Together, these data indicate that GADD45β does not affect the early phase of NF-κB activation but restricts sustained p65 nuclear localization during late necroptotic stages. Despite this reduction in NF-κB signaling, GADD45β did not alter short-term cell viability (Fig. 5a), suggesting that its primary role is to modulate inflammatory responses rather than determine cell death. After 18 h of necroptotic stimulation, GADD45β-expressing cells displayed improved survival compared to controls, indicating a potential protective role for GADD45β in long-term resistance to necroptosis (Fig. 5b). This survival advantage was abolished by treatment with the RIPK3 inhibitor GSK-872 (Fig. 5c), confirming that cell death under these conditions was RIPK3-dependent. In contrast, treatment with the global NF-κB inhibitor IKK-16 led to robust caspase activation, which was fully suppressed by the pan-caspase inhibitor zVAD. Notably, GADD45β expression did not exacerbate apoptosis induced by complete NF-κB inhibition, indicating that it does not sensitize cells to caspase-dependent death (Fig. 5d). In conclusion, GADD45β selectively attenuates RIPK3-driven inflammatory signaling without amplifying apoptotic responses associated with complete NF-κB suppression.

### Inducible GADD45β expression dampens IKK-dependent CXCL8 production during necroptosis

**Fig. 6:**
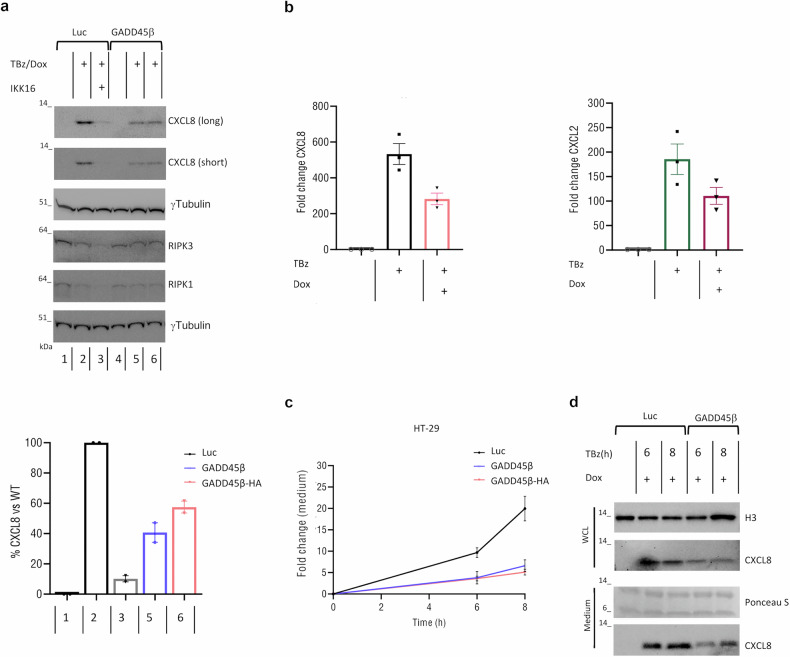
GADD45β modulates CXCL8 expression during necroptosis. **a** Immunoblot of CXCL8 and indicated proteins in HT-29 Luc and GADD45β cells treated as indicated for 7 h. CXCL8 quantified relative to maximal Luc response (black), with Dox-treated GADD45β (blue) or GADD45β-HA (pink). The means of two independent experiments are displayed as bars. **b** Relative CXCL8 and CXCL2 mRNA levels in HT-29 cells after 7 h necroptotic stimulation, quantified by qRT-PCR normalized to GAPDH. The means of three independent experiments (*n* = 3) are displayed as bars. Values of each independent experiments are shown as points. **c** CXCL8 secretion measured at indicated times by Promega Lumit IL-8 immunoassay. Fold changes were calculated relative to the baseline condition (untreated control) and compared between Dox-treated GADD45β (blue), GADD45β-HA (pink), and Luc control cells (black). Each data point represents the mean of two independent experiment; error bars show the range. **d** HT29 Luc and GADD45β cells were stimulated with TBz for 6 and 8 h. The supernatants and cell lysates were collected and analysed by western blotting. Ponceau S was used as a loading control for the supernatants. Representative immunoblots shown.

To address whether GADD45β exerts a broad or selective effect on NF-κB-dependent proinflammatory mediators during necroptosis, we measured the expression of several canonical NF-κB target genes, including the chemokines CXCL8 and CXCL2, the proinflammatory cytokine IL1B, and the adhesion molecule ICAM1, in HT-29 cells expressing inducible GADD45β following TBz stimulation. While CXCL8 and CXCL2 showed significant upregulation in control cells and were markedly reduced upon GADD45β induction (Fig. 6a, b), expression levels of IL-1beta and ICAM1 remained largely unchanged (Supplementary Fig. S4a, b). Similarly, treatment with the IKK inhibitor IKK-16 abolished TBz-induced CXCL8 expression, confirming that IKK complex activation is required for CXCL8 expression during necroptosis (Fig. 6a).

Time-course analysis revealed a sustained increase in CXCL8 secretion during necroptosis, which occurred in parallel with the phosphorylation of RIPK3 and MLKL (Supplementary Fig. S4c, d), consistent with a robust inflammatory response. However, doxycycline-induced expression of GADD45β prior to TBz treatment significantly attenuated CXCL8 secretion (Fig. 6c, d). These results identify GADD45β as a selective inhibitor of RIPK3-dependent cytokine signaling, specifically limiting CXCL8 expression and secretion via IKK-dependent mechanisms. This underscores a context-specific role for GADD45β in restraining chemokine-driven inflammatory responses during necroptosis.

## Discussion

Addressing the previously unexplored question of whether GADD45β directly modulates RIPK3-driven processes, our study identifies GADD45β as a novel regulator of necroptotic signaling. We provide compelling evidence that GADD45β binds directly to RIPK3 in a RHIM-independent manner, interfering with RIPK1-RIPK3 complex formation and attenuating RIPK3-driven NF-κB activation. This interaction suppresses inflammatory outputs, such as CXCL8 and CXCL2 production, without significantly altering early cell viability. Importantly, we show that GADD45β confers a protective advantage in long-term necroptotic conditions, and this effect is reversed by the RIPK3 inhibitor, confirming the specificity of its action in RIPK3-dependent necroptosis. In parallel, we demonstrate that global inhibition of NF-κB by the IKK inhibitor induces strong caspase activation, which is blocked by zVAD. Notably, GADD45β expression does not exacerbate apoptosis under these conditions, indicating that it selectively modulates RIPK3-mediated signaling without promoting caspase-dependent cell death. These findings position GADD45β as a non-redundant regulator of necroptosis-associated inflammation, rather than a general suppressor of NF-κB or cell viability.

Mechanistically, our data support a model in which GADD45β acts as a regulatory checkpoint that fine-tunes RIPK3 signaling under inflammatory stress. RIPK3 is known to mediate MLKL activation and necroptotic cell death, and it also promotes transcriptional activation through NF-κB signaling, particularly under sublethal or low-RIPK3 expression conditions [20–22]. Our data indicate that GADD45β antagonizes the latter function. We identify a multiprotein complex comprising NEMO, IKK, RIPK3, and RIPK1, which suggests the existence of a signaling axis linking necroptosis to canonical NF-κB activation. This aligns with previous reports indicating that necroptosis can promote cell-autonomous cytokine expression via IKK-dependent mechanisms [40].

In support of this, we find that GADD45β selectively limits NF-κB-dependent transcription of chemokines such as CXCL8 and CXCL2, without significantly impacting other NF-κB targets such as IL1B and ICAM1. This transcriptional specificity highlights the ability of GADD45β to shape the inflammatory signature associated with RIPK3 activation. Time-course experiments further show that GADD45β suppresses sustained CXCL8 secretion following RIPK3 activation, emphasizing its role in controlling necroptosis-driven inflammation.

Notably, the effects of GADD45β are in line with emerging models of sublethal necroptosis, in which partial activation of RIPK3 promotes sustained NF-κB signaling, whereas its complete activation toward full necroptosis leading to a more immunogenic tumor environment [23, 24]. Our data imply that GADD45β could serve to retain RIPK3 in a sublethal, non-lytic state, thereby blocking necroptotic cell death. This positions GADD45β as a critical modulator and therapeutic candidate in RCD-driven pathologies. Importantly, recent studies have demonstrated that disruption of RIPK1 scaffolding can synergistically promote necroptosis and innate immune activation, fostering tumor immunogenicity [41]. Consistent with its broader immunomodulatory functions, GADD45β has been implicated in tumor-associated macrophages reprogramming and CD8 + T-cell recruitment in hepatocellular carcinoma [31]. Our findings extend this role by identifying RIPK3 as a novel interacting partner. Although RIPK3 is silenced in several tumor types via promoter hypermethylation [42], it remains expressed and functional in others [23, 43], making necroptosis a viable target in specific contexts.

A limitation of our study is that the functional impact of GADD45β-RIPK3 interaction has been primarily assessed in cell lines; Future studies should explore whether GADD45β exerts similar regulatory functions in primary immune cells or in vivo models of necroptosis-driven inflammation and cancer [44]. Additionally, dissecting how GADD45β interfaces with other RIPK3-binding partners could shed light on broader regulatory networks governing inflammatory cell death. Structural studies will also be essential to resolve the interface and dynamics of the GADD45β-RIPK3 interaction and to assess the feasibility of targeting this axis for therapeutic purposes. In summary, our results establish GADD45β as a selective and context-specific modulator of RIPK3-driven inflammatory signaling, able to dampen chemokine production and protect against necroptotic death without triggering compensatory apoptosis.

## Materials and methods

### Reagents and antibodies

Recombinant human TNFα (Cat# 210-TA) and IKK-16 (Cat# 2539) were obtained from Bio-Techne (Minneapolis, MN, USA), GSK-872 (Cat# S8465) from Selleck Chemicals (Houston, TX, USA), zVAD (Cat# 102265357) from Sigma (Burlington, MA, USA), and BV6 (Cat# INH-BV6) from InvivoGen (San Diego, CA, USA). Primary antibodies used for immunoblotting included those against: GADD45β (MyBioSource, Cat# MBS821452), RIPK1 (BD Biosciences, Cat# 610459), RIPK3 (MyBioSource, Cat# MBS2112394), FLAG (Sigma, Cat# F3165-2MG), IKKγ (Abcam, Cat# AB178872), FADD (Santa Cruz Biotechnology, Cat# sc-6036; Enzo Life Sciences, Cat# ADI-AAM212), α-Tubulin (Abclonal, Cat# AC012), γ-Tubulin (Sigma, Cat# T6557-2ML), Histone H3 (Abclonal, Cat# A2348), IKKα/β (Santa Cruz Biotechnology, Cat# sc-7607), IκBα and phospho-IκBα (Ser32) (Cell Signaling Technology, Cat# 9242S and 9246), HA (Santa Cruz Biotechnology, Cat# sc-805), CXCL8 (ProteinTech, Cat# 17038-1-AP), NF-κB p65 (Abclonal, Cat# A2547) and Phospho-NF-kB p65/RelA-S536 (Abclonal, Cat# AP1294). HRP-conjugated secondary antibodies were from Bio-Rad (Cat# 172-1011, 170-6515).

### Biolayer Interferometry (BLI) label-free binding assays

The BLI technique was used to assess the binding of *rh*GADD45β [45] towards the human recombinant RIPK3 (*rh*RIPK3; Mybiosource cod. MBS1442585). AR2G sensor chip (Sartorius) was functionalized using RIPK3 at 10 μg/mL in 10 mM NaAc at pH 4.5 according to the manufacturer’s instructions. A reference channel used as blank was properly prepared. The *rh*GADD45β was tested at reported increased concentrations using 25 mM Tris pH = 7.5 containing 100 mM NaCl, 0.02% Tween 20, 0.1% BSA as running buffer. All analyses were carried out at 25 °C, and 5 mM NaOH was used to regenerate the chip surface. All mathematical manipulations and fitting were performed using the Octet Analysis Studio 12.2 from Sartorius. Data were fitted assuming a 1:1 Langmuir binding model. Binding curves were exported and charted using GraphPad (GraphPad Software Inc., San Diego, CA, USA).

### Cell culture and transfection

HEK293T and HT29 cells were cultured in DMEM or McCoy’s 5 A Modified medium (GIBCO), respectively, supplemented with 10% FBS, 50 U/mL penicillin, and 50 µg/mL streptomycin. Cells were maintained at 37 °C in a humidified 5% CO2 incubator. HEK293T cells were transfected using Lipofectamine 3000 (Invitrogen) per the manufacturer’s instructions. HEK293-NEMO-KO cells were previously described [46]. HT29 cells (ATCC Cat# HTB-38) were treated with IKK-16 and BV6 (1 µM), zVAD (20 µM), and TNFα (10 or 30 ng/mL). All cell lines were routinely confirmed Mycoplasma-free using PCR-based assays.

### FLAG immunoprecipitation (IP) and Western blotting

Cells were lysed in buffer containing 120 mM NaCl, 2 mM KCl, 10% glycerol, 2 mM EDTA, 1% Triton X-100, and 30 mM Tris-HCl, pH 7.4, supplemented with protease inhibitors. Lysates were clarified by centrifugation (14,000 rpm, 10 min, 4 °C). Protein concentrations were determined by BCA assay. For co-IP, extracts were incubated with anti-FLAG agarose beads (Sigma-Aldrich) for 4 h at 4 °C, washed 5x in lysis buffer, and subjected to SDS-PAGE. Nuclear (NE) and cytoplasmic (CE) protein extracts were prepared using the NE-PER™ Nuclear and Cytoplasmic Extraction Reagents kit (Thermo Fisher Scientific, Cat# 78833) following the manufacturer’s protocol. Western blots were transferred to nitrocellulose membranes, blocked in 5% milk (or BSA for primary antibody incubation), and probed overnight with primary antibodies. Detection was performed with ECL (Pierce) and visualized with X-OMAT film on a ChemiDoc Touch Imaging System (Bio-Rad). Uncropped western blots are included as Supplementary Data.

### Luciferase reporter assays

HEK293T cells were transfected with reporter plasmids including NF-κB-luciferase (κB-Luc, 200 ng) and indicated constructs. Total DNA per well was normalized with empty vector. Doxycycline-induced expression of GADD45β was performed at indicated concentrations. Luciferase activities were assessed 24 h later, in a dual luciferase assay (Promega Cat# E2920). Data were reported as the ratio of firefly arbitrary units for each sample to firefly arbitrary units for the sample with only the κB-Luc (RLU).

### Cell viability assay

HT29 cells were seeded at 4 × 10^3^ cells/well in 96-well plates. After 24 h, cells were pretreated with zVAD (20 µM), BV6 (1 µM), GSK-872 (5 µM) or DMSO for 1 h, followed by TNFα (30 ng/mL) for the period indicated in the figures. Viability was assessed using CellTiter-Glo (Promega, Cat# G7570) and Caspase-3/7 activity was measured using the Caspase-Glo 3/7 kit (Promega, Cat# G8090), on a Victor PerkinElmer luminometer. Luminescence was measured using a Victor PerkinElmer 96-well plate reader.

### Plasmid construction

The inducible Tet-one system was used to construct an inducible expression vector of GADD45β and HA-tagged GADD45β at the C-terminus by PCR amplification from the cDNA from the pM-C HA mGADD45β (ABM Richmond, British Columbia, Canada. Cat# 211750240500). PCR products were inserted between the BamHI and EcoRI sites of the pLVX-TetOne-puro vector. The pLVX-TetOne-Luciferase lentivirus vector was used as control.

### Lentiviral packaging and cell transduction

HEK293FT cells were transfected with psPAX2, pCMV-VSV-G (Addgene), and lentiviral vectors (2:1:5 ratio) using Lipofectamine 3000. Virus-containing supernatants were collected 48 h post-transfection and used to infect target cells with 5 µg/mL polybrene. Infected cells were selected with 2 µg/mL puromycin for 5–7 days. Lentivirus particles were prepared using pLVX-TetOne-Luciferase and pLVX-TetOne- GADD45β or GADD45β -HA plasmids.

### Quantitative PCR (qPCR) and semi-quantitative reverse transcription PCR (RT-PCR) analysis

Total RNA was extracted from HT29 cells using the RNeasy Mini Kit (Qiagen, Venlo, Netherlands) with on-column DNase treatment. cDNA was synthesized from 1 µg RNA using SuperScript IV VILO (Invitrogen). Gene expression was quantified using SYBR Green (Applied Biosystems) on a 7900HT system. Primers used included:

GADD45β (5’TCCTCAGCGTTCCTCTAGA3’; 5’AATTCATGACCCTGGAAGAGCTG-3’);

GAPDH (5’-TTGCCATCAATGACCCCTTCA-3’; 5’-CGCCCCACTTGATTTTGGA-3’);

CXCL8 (5’-CAGTTTTGCCAAGGAGTGCT-3’; 5’-ACTTCTCCACAACCCTCTGC-3’);

CXCL2 (5’-CACTCAAGAATGGGCAGAAAG-3’; 5’-TCAGGAACAGCCACCAATAAG-3’)

ICAM (5’ – GAACCAGAGCCAGGAGAC – 3’; 5’- CATTCAGCGTCACCTTGG – 3’)

IL-1beta (5- ATGATGGCTTATTACAGTGGCAA -3; 5- GTCGGAGATTCGTAGCTGGA -3’)

### Immunocytochemistry

HT-29 cells were seeded on gelatin-coated slides. After 24 h, cells were treated with inhibitors and TNFα as described, then fixed with 4% PFA. Samples were incubated with primary antibodies in BSA solution (0.5% BSA, 0.05% Saponin, 0.02% NaN_3_, 50 mM NH_4_Cl, PBS), overnight at 4 °C, followed by Alexa Fluor 488 secondary antibodies (Life Technologies, Cat# A11008) and Hoechst 33348 nuclear staining (ThermoFisher). Samples were mounted with SlowFade Gold Antifade Reagent (ThermoFisher Cat #S36937).

### Lumit immunoassays

The immunoassay for IL-8 was performed using the Lumit™ IL-8 (human) (Promega Cat# CS2032C02). Prior to the immunoassay, 5000 cells were seeded in 100 µl of McCoy’s 5 A Modified growth medium in a 96-well plate and incubated overnight. The following day, the medium was replaced, and the cells were pre-treated with zVAD (20 μM), BV6 (1 μM), or DMSO for 30 min, after which they were stimulated with TNF (20 ng/mL) for the indicated time. After treatment, 50 µL of medium was transferred to a new plate and incubated for 90 min with a mixture of Anti-hIL-8 mAb-SmBiT and Anti-hIL-8 mAb-LgBiT antibodies at a 2X concentration. In the same plate, this antibody mixture was incubated with only medium and known concentrations of IL-8 obtained through serial dilutions of the human IL-8 standard provided by the kit. Following incubation, the Lumit™ Detection Reagent was prepared by diluting Lumit™ Detection Substrate B 1:20 in Lumit™ Detection Buffer B supplied with the kit. After a 5-min incubation, luminescence was measured using a 96-well plate reader (Victor PerkinElmer microplate luminometer).

### Quantification and statistical analysis

The analyses were conducted using GraphPad Prism v10 (San Diego, CA, USA). Sample sizes (*n*) for each experiment were based on feasibility and established laboratory practice. For experiments with *n* < 5, individual data points from independent experiments are shown in all figures. For *n* = 2, no descriptive or inferential statistics were calculated. Individual data points or means are shown to allow transparent visualization of data variability. For experiments with *n* ≥ 3, descriptive statistics (mean ± SEM) and appropriate inferential tests (e.g., two-tailed unpaired Student’s *t* test or ANOVA with post hoc corrections) were conducted as indicated in the figure legends. To establish the percentage of cells with p65 nuclear localization, we used DAPI staining to specifically delimitate the nuclear compartment for each cell. Nuclear Integrated Density (IntDen) from p65 (green) channel was measured in the nuclear area of each cell by using ImageJ software (National Institutes of Health, Bethesda, MD, USA). The highest nuclear IntDen value in the appropriate control condition (untreated, NT, Luc or NT GADD45β) was used to establish the threshold beyond which a cell is considered positive for nuclear p65 (when nuclear IntDen>Threshold, the individual cell is considered positive for nuclear p65). Percentage of cells positive for nuclear p65 was calculated for each field. The analysis was performed in two independent experiments. 7–18 fields per condition (68–152 cells, Fig. 4C) or 6–9 fields per condition (21–38 cells, Supplementary Fig. S3) were analysed. Each data point from the graphs represents the percentage of cells positive for nuclear p65 in one field. Data are shown as mean ± SEM. Statistical analyses were conducted using GraphPad Prism. Significance was determined after outliers removal (alpha = 0.05). In case of not fulfilling homoscedasticity or normality, square root data correction was performed. When data were homoscedastic and followed a normal distribution, Two-Way ANOVA with Tukey’s post hoc test was employed. In case of not fulfilling homoscedasticity or normality (in spite the square root correction), no parametric Kruskal-Wallis test was used. Significance: *p < 0.05; **p < 0.01; ***p < 0.001; ****p < 0.0001; ns, not significant (*p* > 0,05).

## Supplementary information

Supplementary Figure legends

Supplementary Figure 1

Supplementary Figure 2

Supplementary Figure 3

Supplementary Figure 4

Uncropped IB

## Data Availability

All data supporting the findings of this study are available within the paper and its supplementary information files, and further inquiries can be directed to the corresponding author.
