# Supplementary material for: GADD45β inhibits RIPK3-mediated NF-κB activation by interfering with NEMO-RIPK1-RIPK3 interactions
Source: Cell Death Discov. 2025 Dec 7;12:41. doi: 10.1038/s41420-025-02894-y (PMC12827253; doi:10.1038/s41420-025-02894-y)
Supplement: Supplementary file 1 — Supplementary Figure legends [file 41420_2025_2894_MOESM1_ESM.pdf]

Supplementary Figure S1

(a) NF-κB luciferase reporter assay in HEK293T cells overexpressing RIPK3 or its mutants (D160N, ΔRHIM). The means of two independent experiments are displayed as bars. Representative immunoblots shown. (b) NF-κB luciferase activity was assessed in HEK293 NEMO-KO (KO) cells under the same conditions as in Figure 3a. Data represent mean ± SEM from four independent experiments; statistical analysis yielded $p = 0.071$. Representative immunoblots shown. (c) Co-IP of Flag-RIPK3 or ΔRHIM mutant with HA-GADD45β in NEMO-KO cells. Representative immunoblots shown.

Supplementary Figure S2

(a) HEK293 WT (WT) and NEMO-KO (KO) cells were transfected with the indicated plasmids, lysates were subjected to anti-Flag immunoprecipitation, immunoblot analysis was performed to identify the indicated proteins. Representative experiment of three is shown. (b) Both HEK293 and HEK293 NEMO-KO cells were transfected with the indicated plasmids, lysates were subjected to anti-Flag immunoprecipitation, immunoblot analysis was performed on input, immunoprecipitated and post-immunoprecipitated lysates to identify the indicated proteins. Representative experiment of three is shown. (c) Cells were transfected with the indicated plasmids and were processed as in (b) and the immunoblot analysis was performed to identify the indicated proteins. Representative experiment of two is shown.

Supplementary Figure S3

(a) Representative confocal microscopy images of HT29 Luc and GADD45β cells treated with TBz (TNF, BV6, zVAD) for the indicated time periods in the presence of Dox (1 μg/mL) and stained for p65 (Alexa Fluor 488, green) and nuclei (DAPI, blue). White arrows indicate cell with nuclear p65. Scale bar: 20 μm. Below, quantification of the percentage of cells with nuclear p65, based on 7-18 fields per condition (68-152 cells per condition). (b) Immunoblot analysis of Whole Cell Lysate

(WCL) from of HT-29 Luc and GADD45β cells treated as in (a) was performed to identify the indicated proteins. (c) Immunoblot analysis of nuclear (NE) and cytoplasmic (CE) fractions from HT-29 Luc and GADD45β cells treated as indicated for the specified time points, probed with antibodies against the indicated proteins. * Indicates not specific band. Representative experiment of two is shown. Data mean ± SEM. Statistical significance: **$p \leq 0.0001$ (Kruskal-Wallis test).

Supplementary Figure S4

(a-b) IL-1beta and ICAM1 mRNA levels in HT-29 cells after 7 h necroptotic stimulation all in presence of 1 μg/mL Dox, quantified by qRT-PCR normalized to GAPDH. The means of three independent experiments (n = 3) are displayed as bars. Values of each independent experiments are shown as points. (c) Immunoblot analysis of indicated proteins in HT-29 Luc and GADD45β cells treated with TBz for indicated times. (d) Luc and GADD45β cells were stimulated with TBz for 8 h, Cell viability was measured using the CellTiter-Glo assay and is expressed as a percentage relative to the untreated (no TBz) control. Data represent mean ± SD from two independent experiments (n = 2).
