# Supplementary figures and images for: GADD45β inhibits RIPK3-mediated NF-κB activation by interfering with NEMO-RIPK1-RIPK3 interactions
Source: Cell Death Discov. 2025 Dec 7;12:41. doi: 10.1038/s41420-025-02894-y (PMC12827253; doi:10.1038/s41420-025-02894-y)

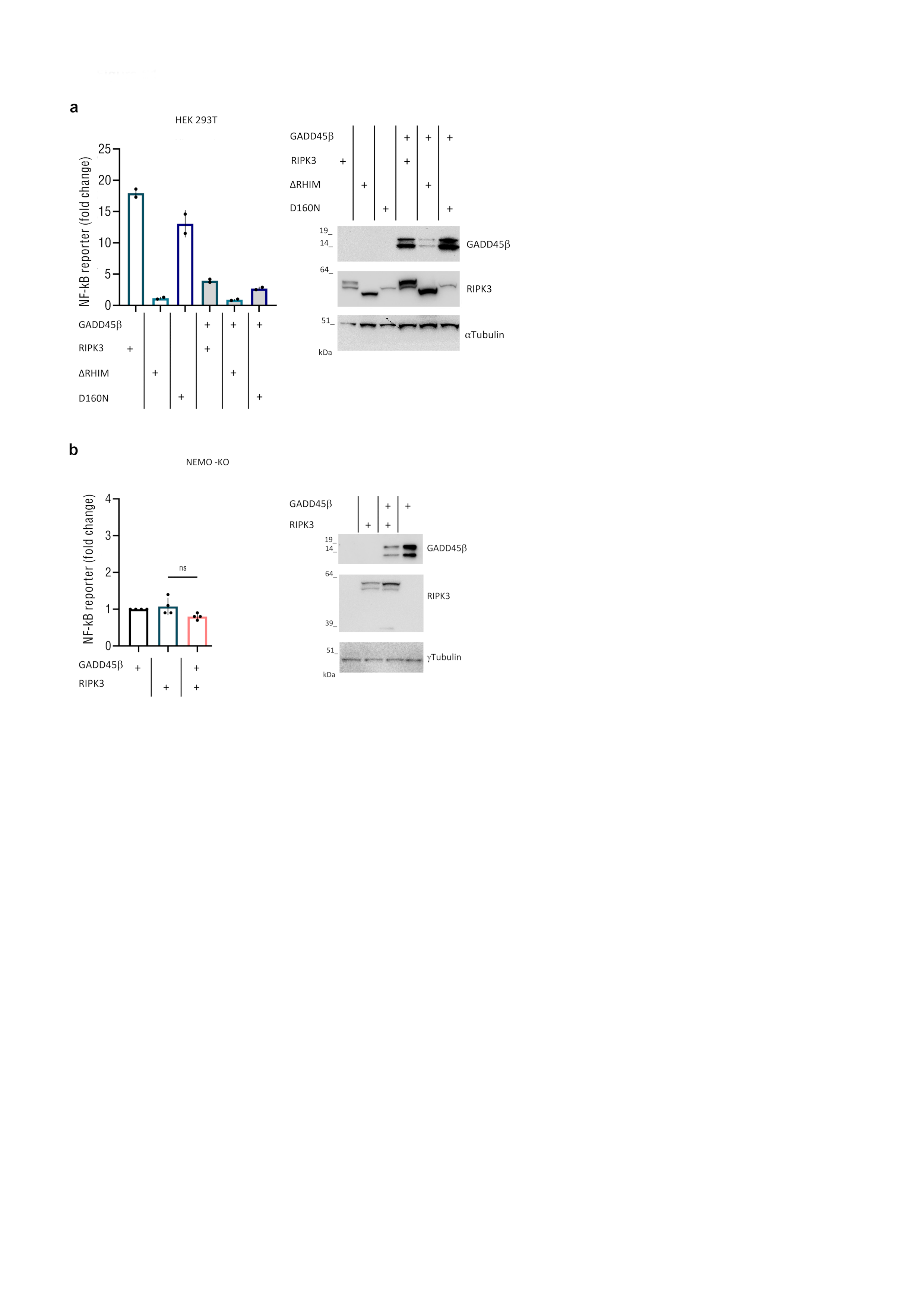

Supplement: Supplementary file 2 — Supplementary Figure 1 [file 41420_2025_2894_MOESM2_ESM.png]

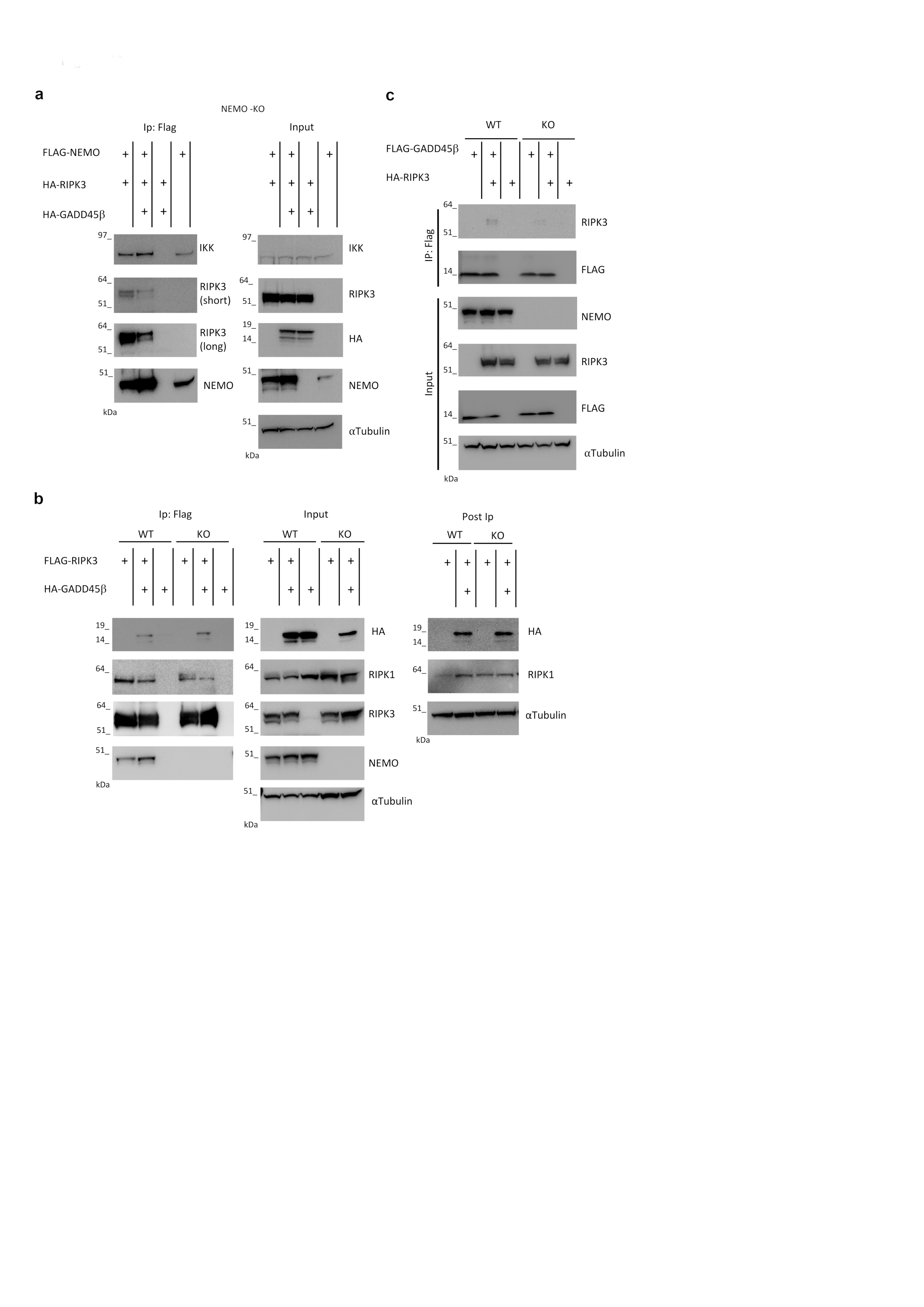

Supplement: Supplementary file 3 — Supplementary Figure 2 [file 41420_2025_2894_MOESM3_ESM.png]

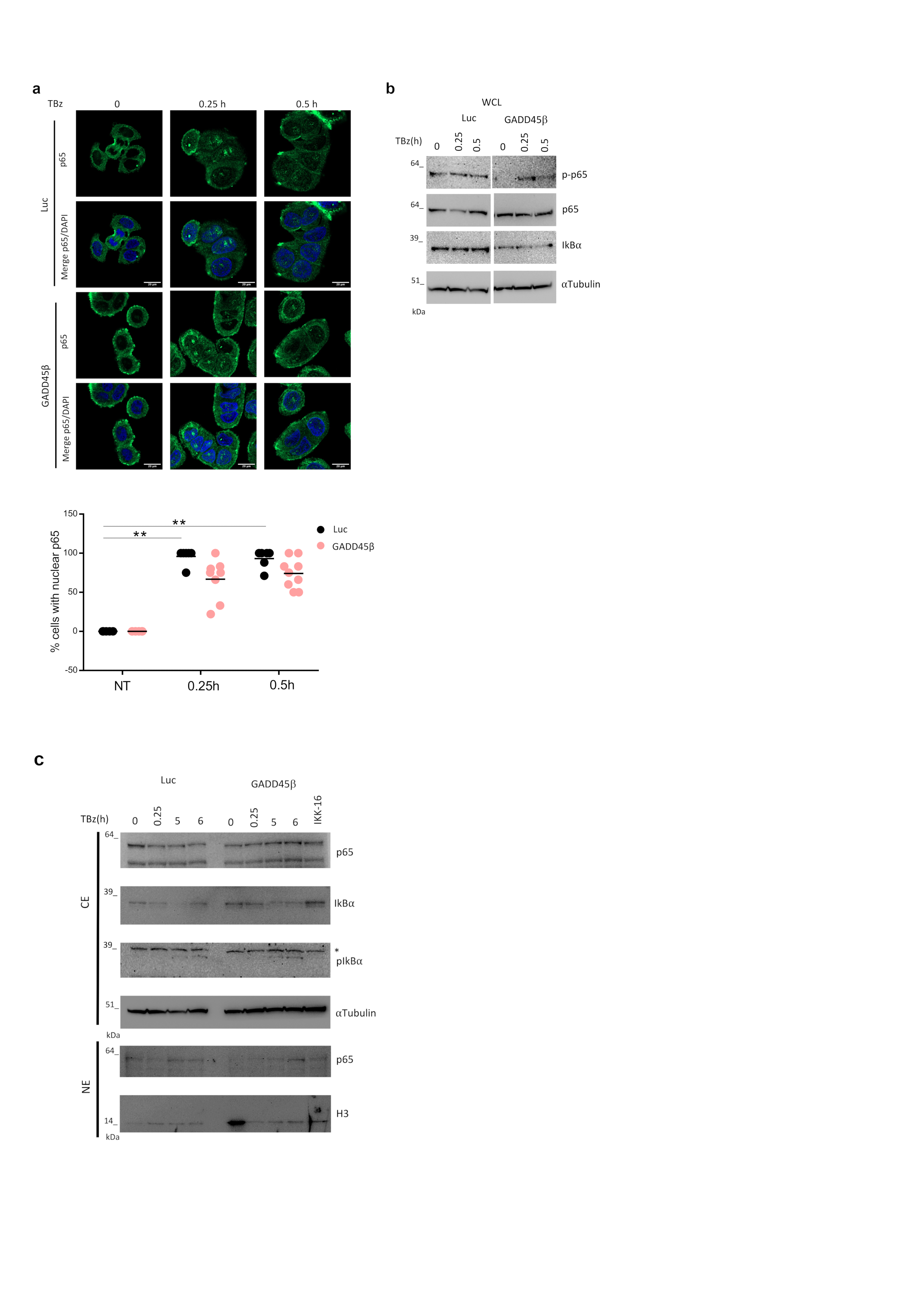

Supplement: Supplementary file 4 — Supplementary Figure 3 [file 41420_2025_2894_MOESM4_ESM.png]

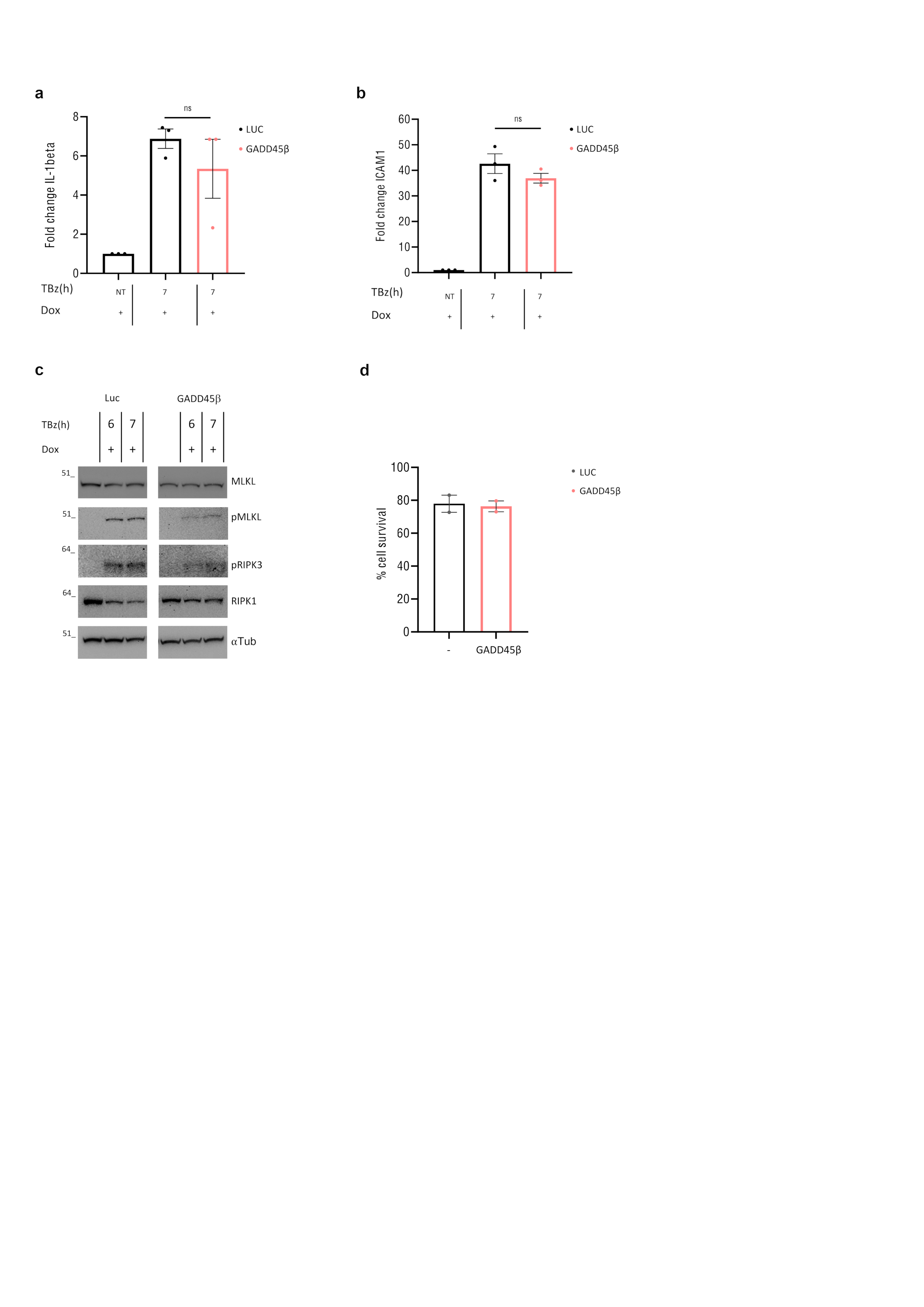

Supplement: Supplementary file 5 — Supplementary Figure 4 [file 41420_2025_2894_MOESM5_ESM.png]
