# Supplementary material for: GADD45β inhibits RIPK3-mediated NF-κB activation by interfering with NEMO-RIPK1-RIPK3 interactions
Source: Cell Death Discov. 2025 Dec 7;12:41. doi: 10.1038/s41420-025-02894-y (PMC12827253; doi:10.1038/s41420-025-02894-y)
Supplement: Supplementary file 6 — Uncropped IB [file 41420_2025_2894_MOESM6_ESM.pdf]

Original data Uncropped IB

Figure 2

a

Input

IP:Flag

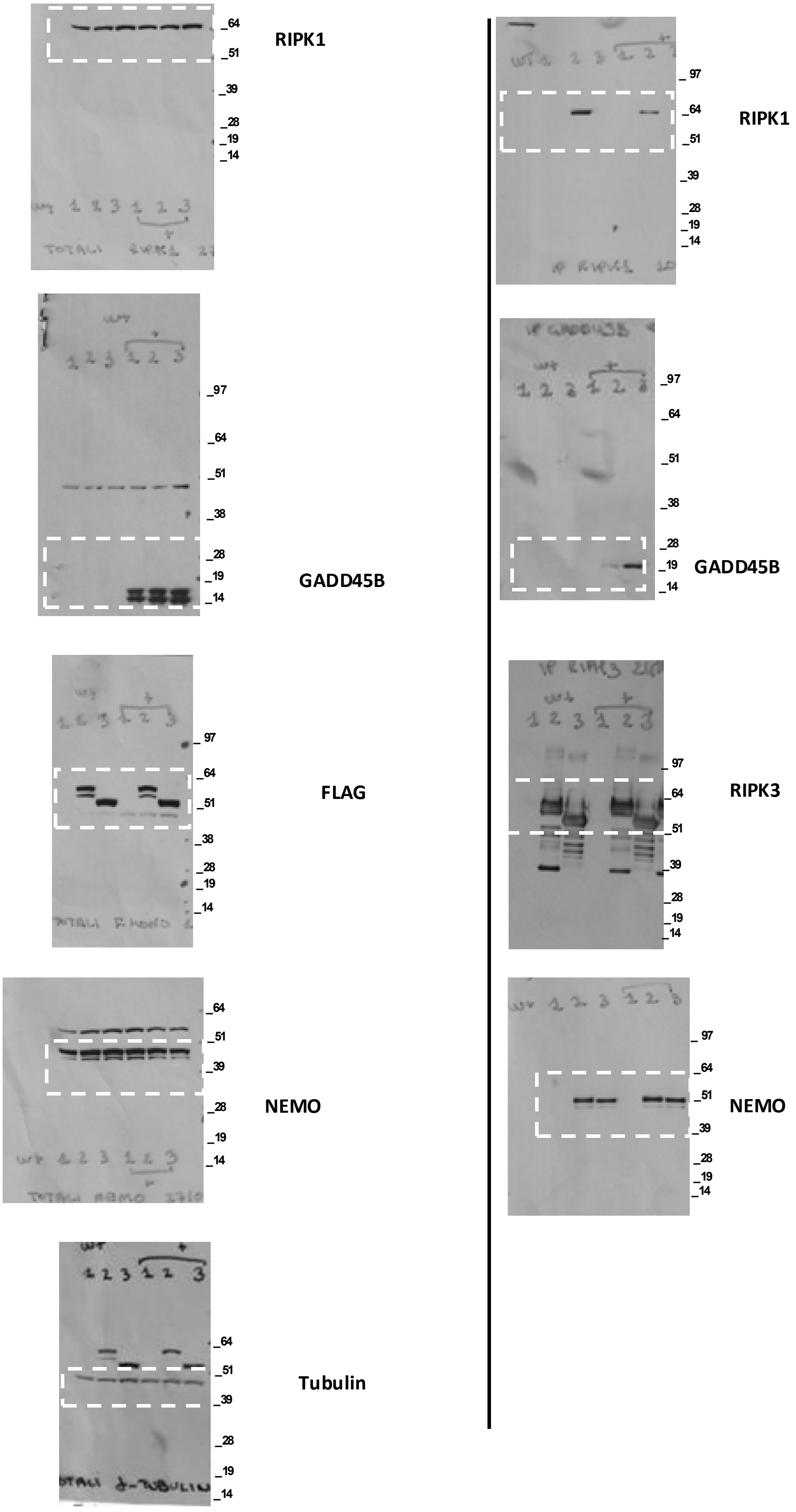

Figure 2 b

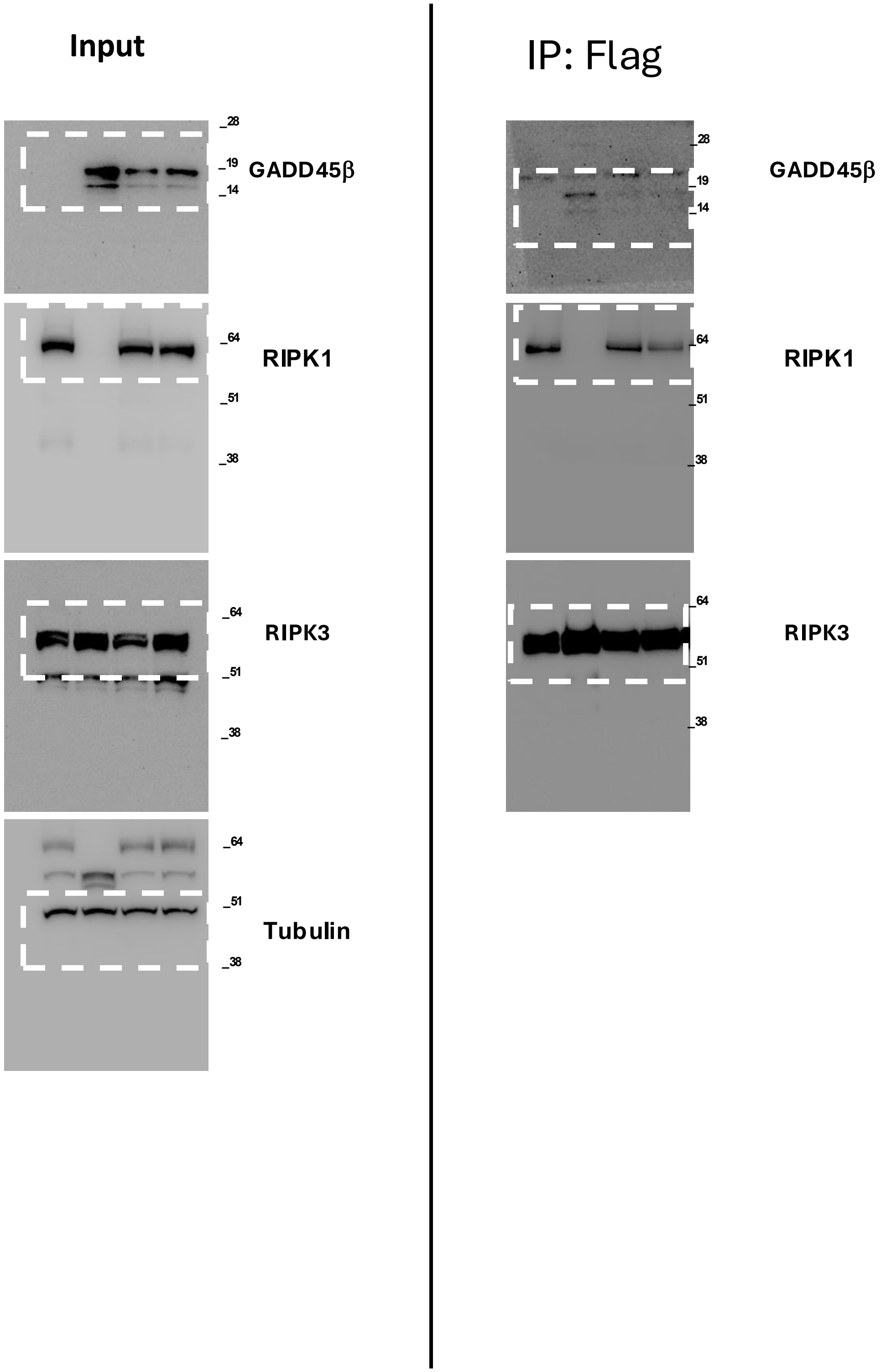

Figure 2

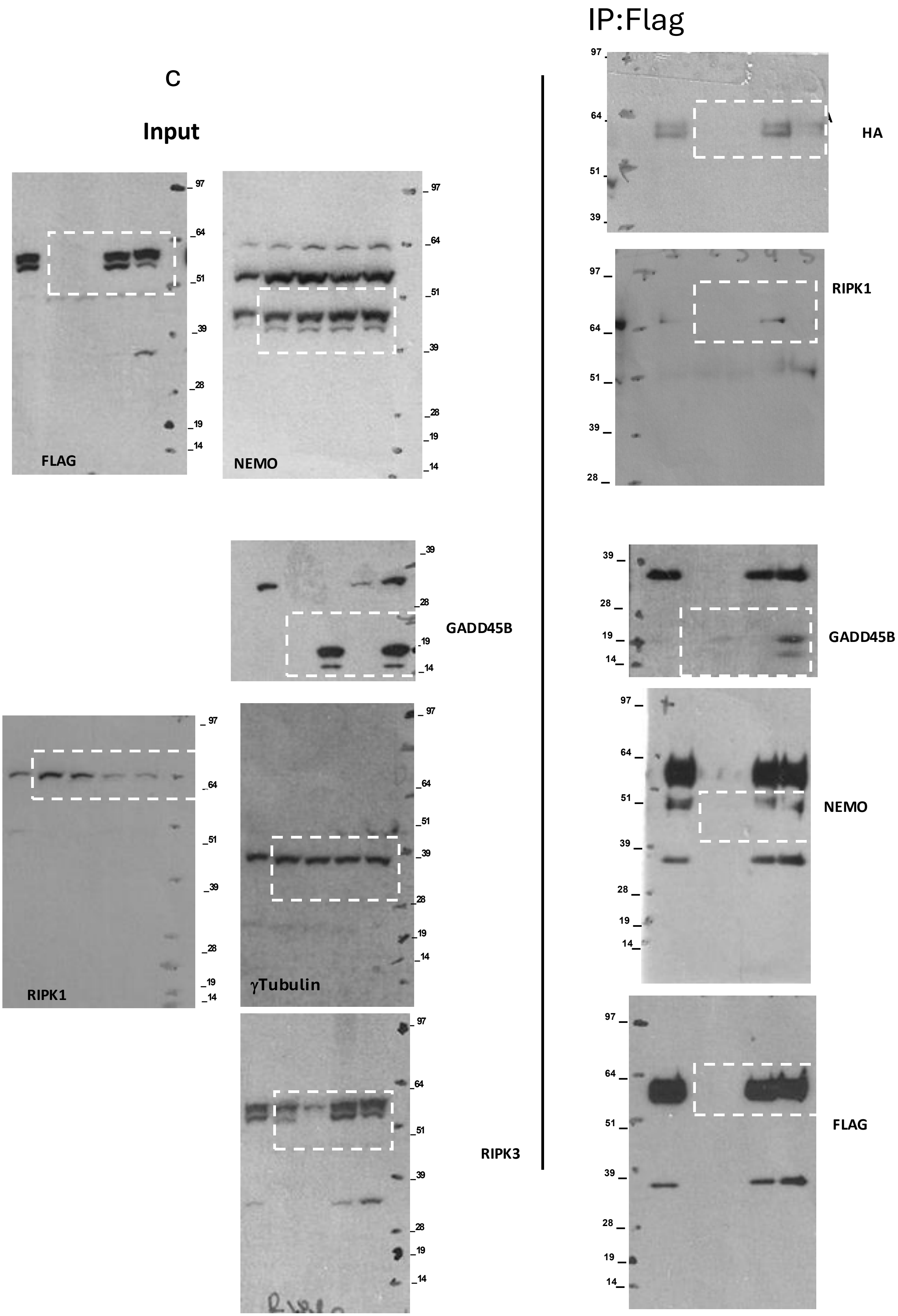

C
Input
IP:Flag
FLAG
NEMO
GADD45B
RIPK1
γTubulin
RIPK3
HA
RIPK1
GADD45B
NEMO
FLAG
97
64
51
39
28
19
14

Figure 3

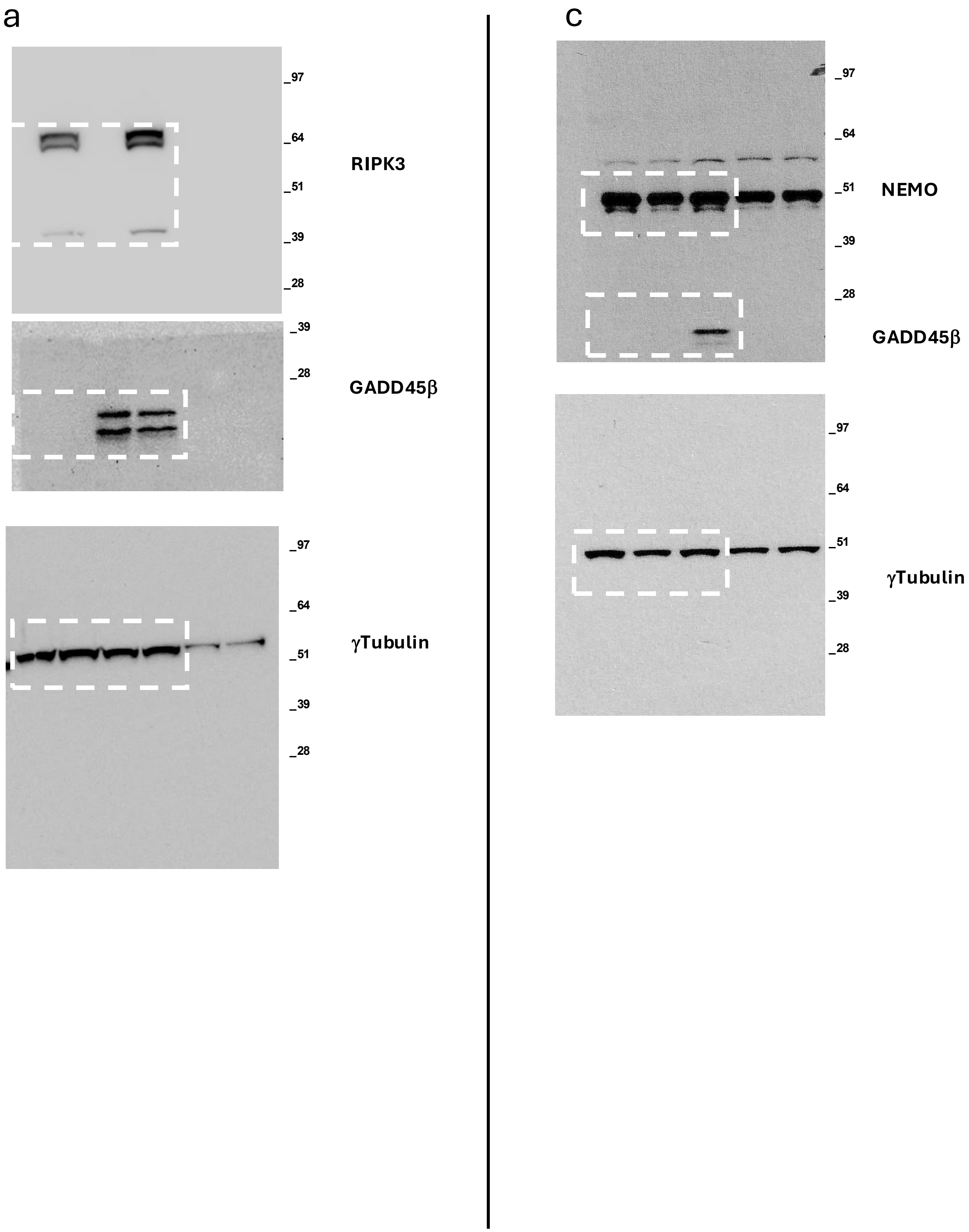

Figure 3b

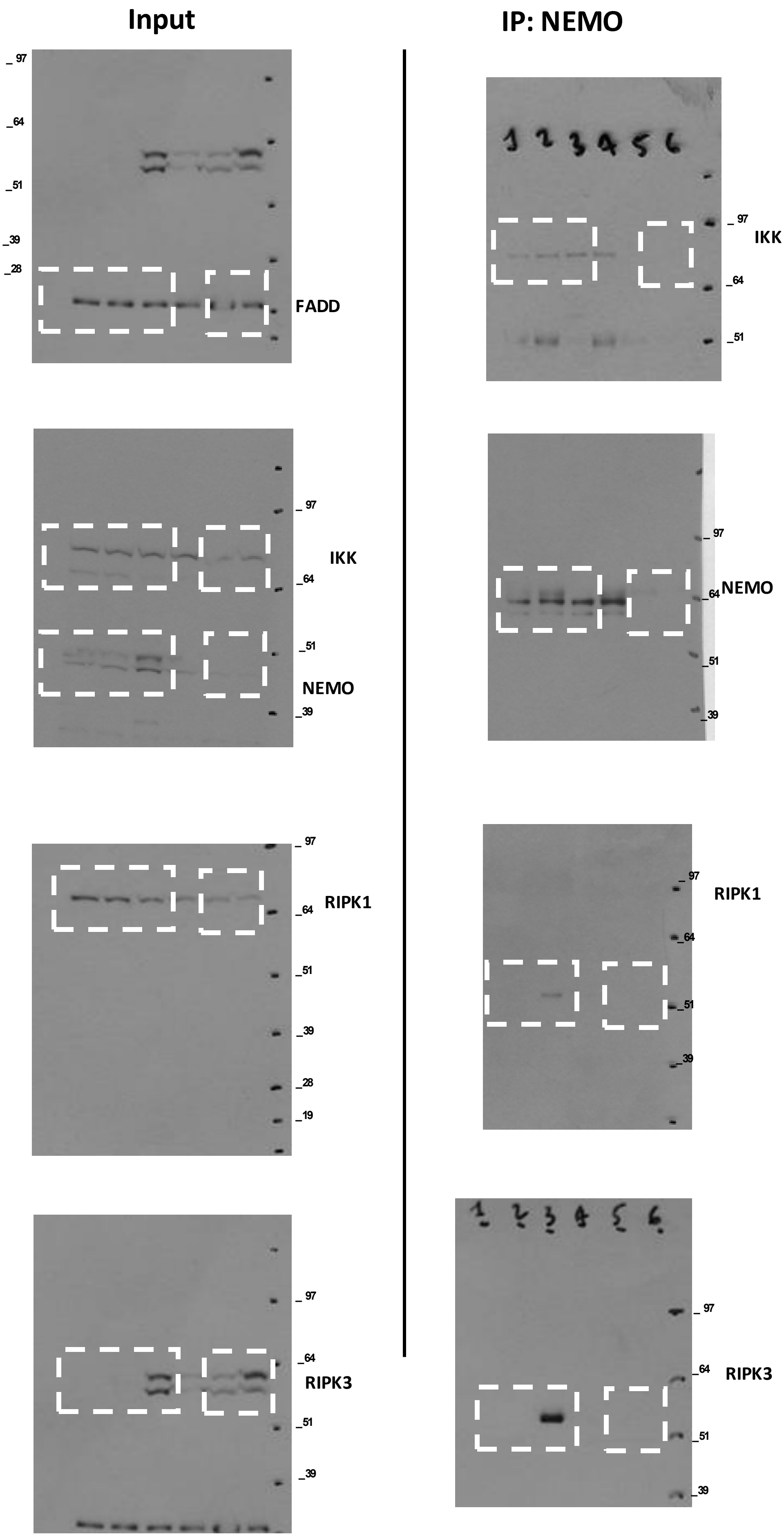
Input
IP: NEMO
97
64
51
39
28
FADD
IKK
NEMO
RIPK1
19
RIPK3
1 2 3 4 5 6

Figure 3

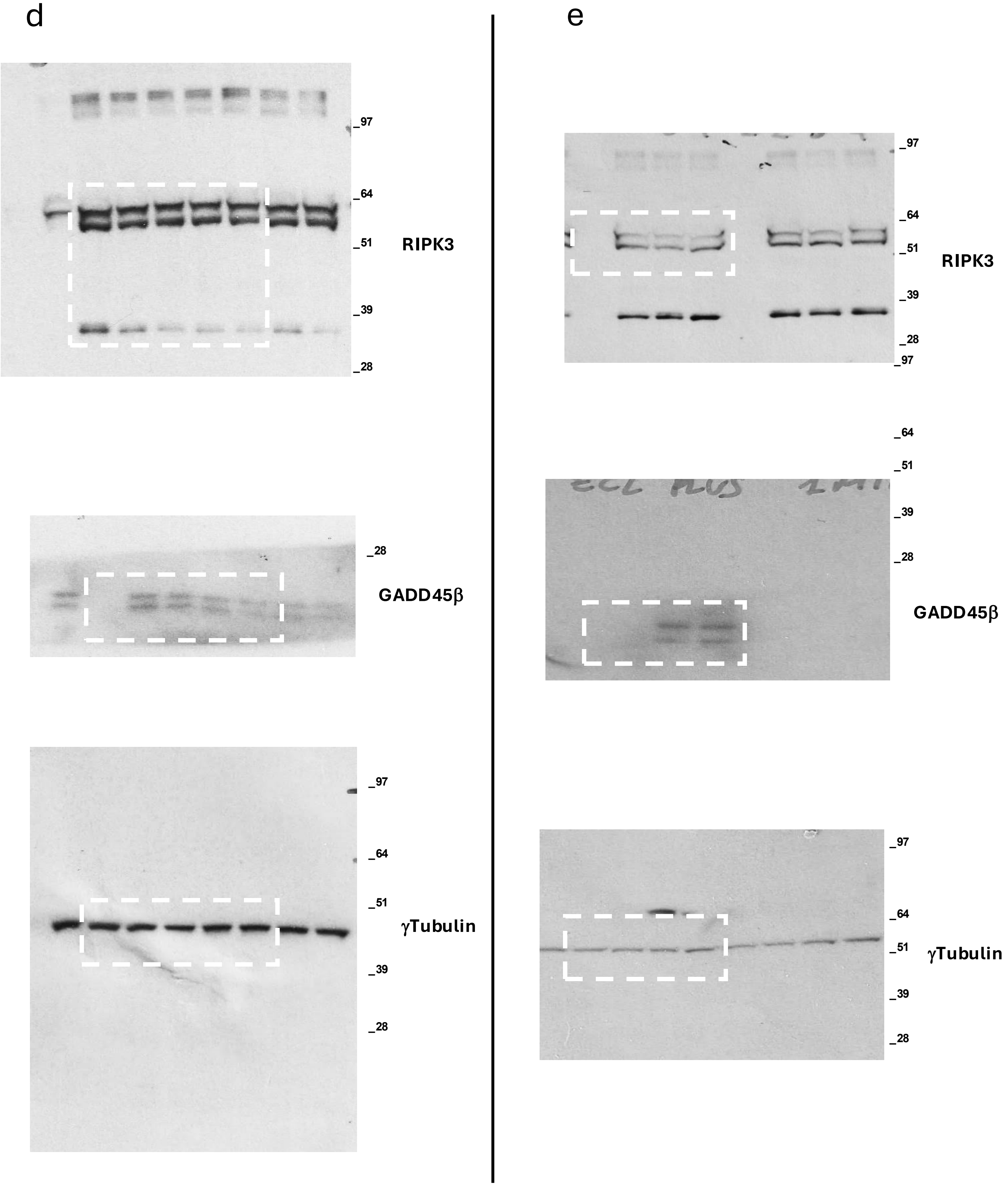

Figure 3

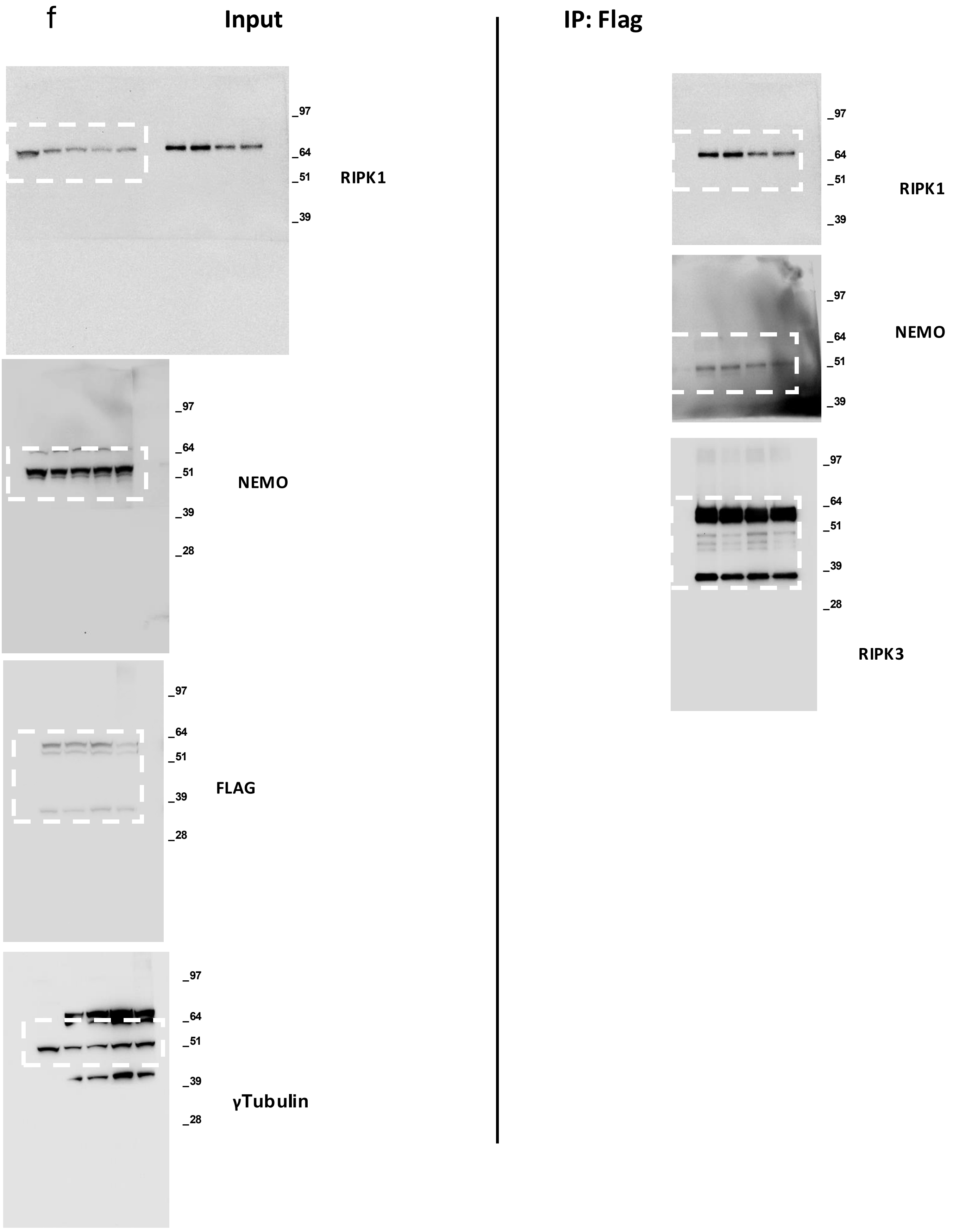

f
Input
IP: Flag
_97
_64
_51
_39
RIPK1
_97
_64
_51
_39
_28
NEMO
_97
_64
_51
_39
_28
FLAG
_97
_64
_51
_39
_28
γTubulin
_97
_64
_51
_39
RIPK1
_97
_64
_51
_39
NEMO
_97
_64
_51
_39
_28
RIPK3

Figure 6 a

Gel 12%

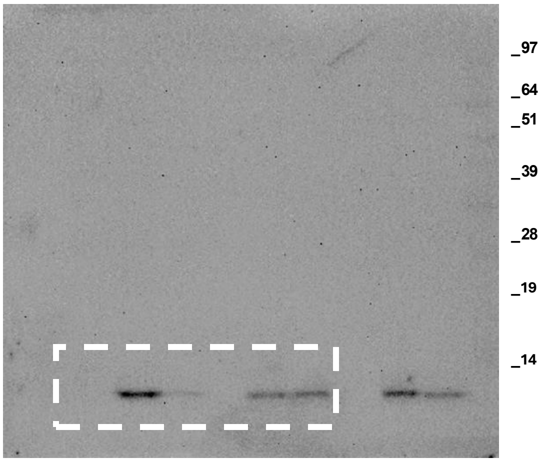

CXCL8 (long)

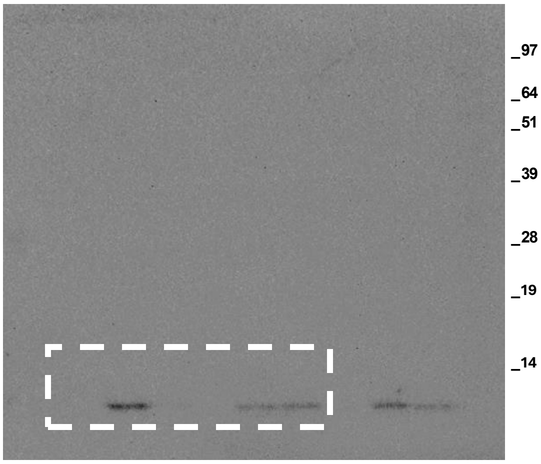

CXCL8 (short)

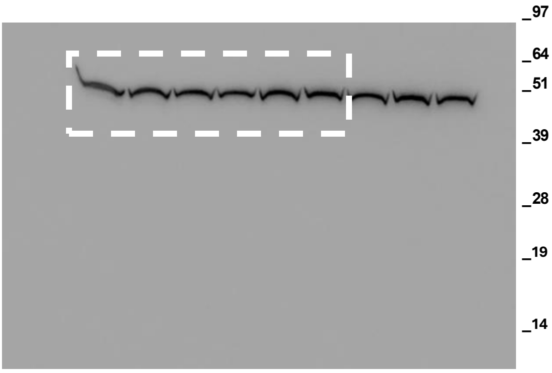

Tubulin

Gel 4-12%

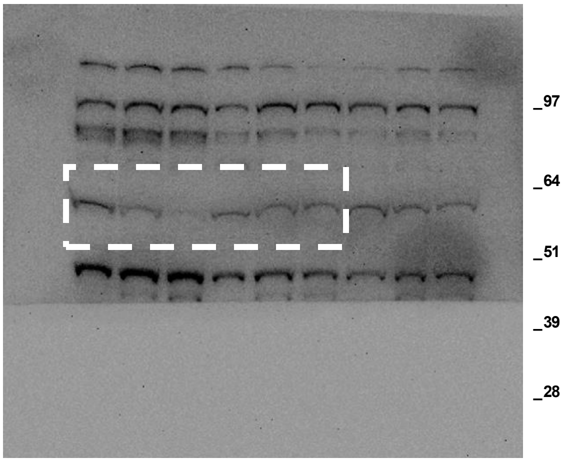

RIPK3

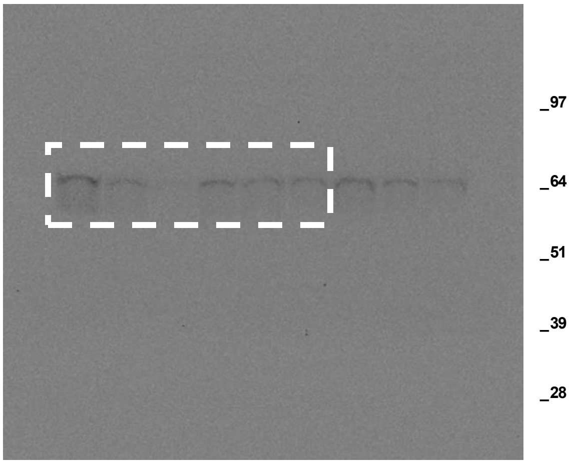

RIPK1

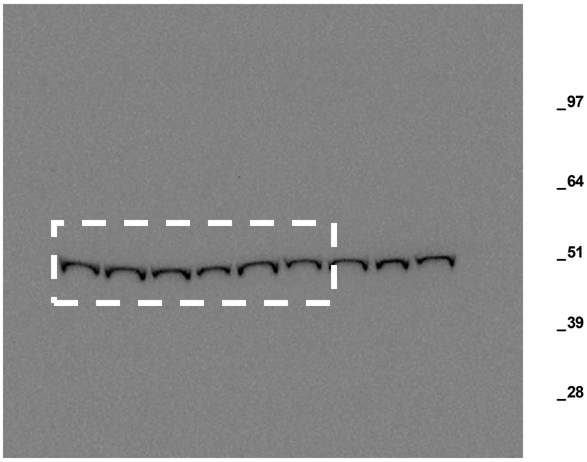

Tubulin

Figure 6d

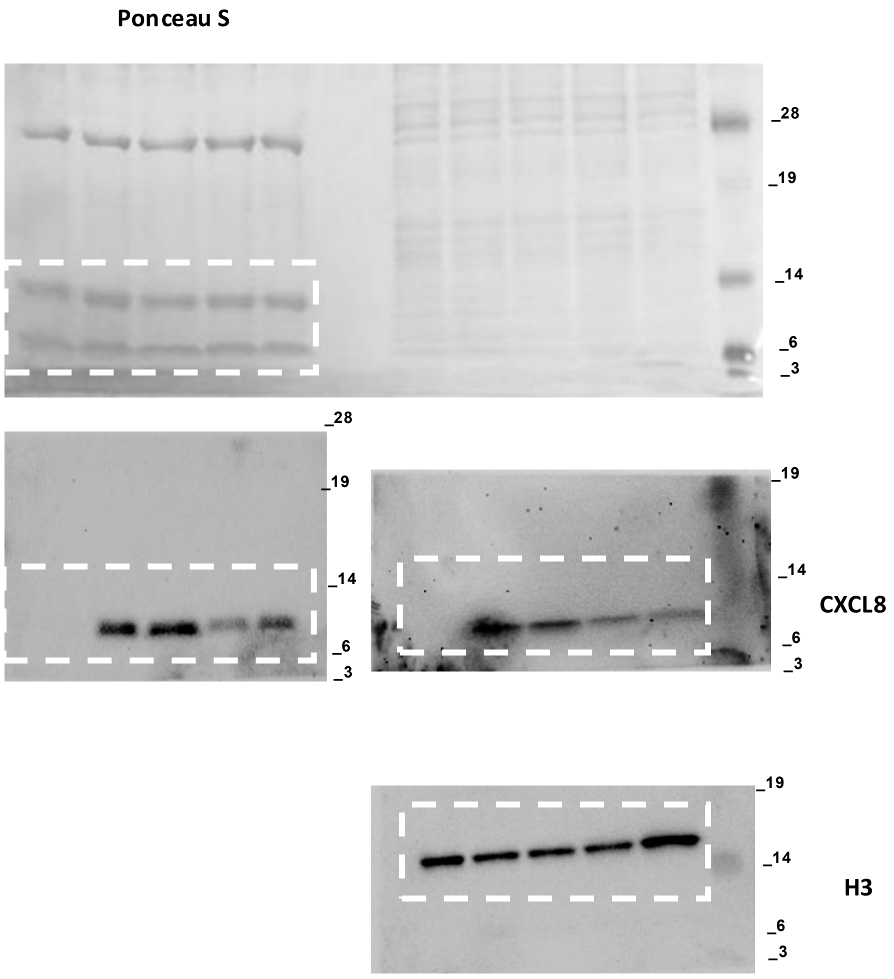

Figure S1

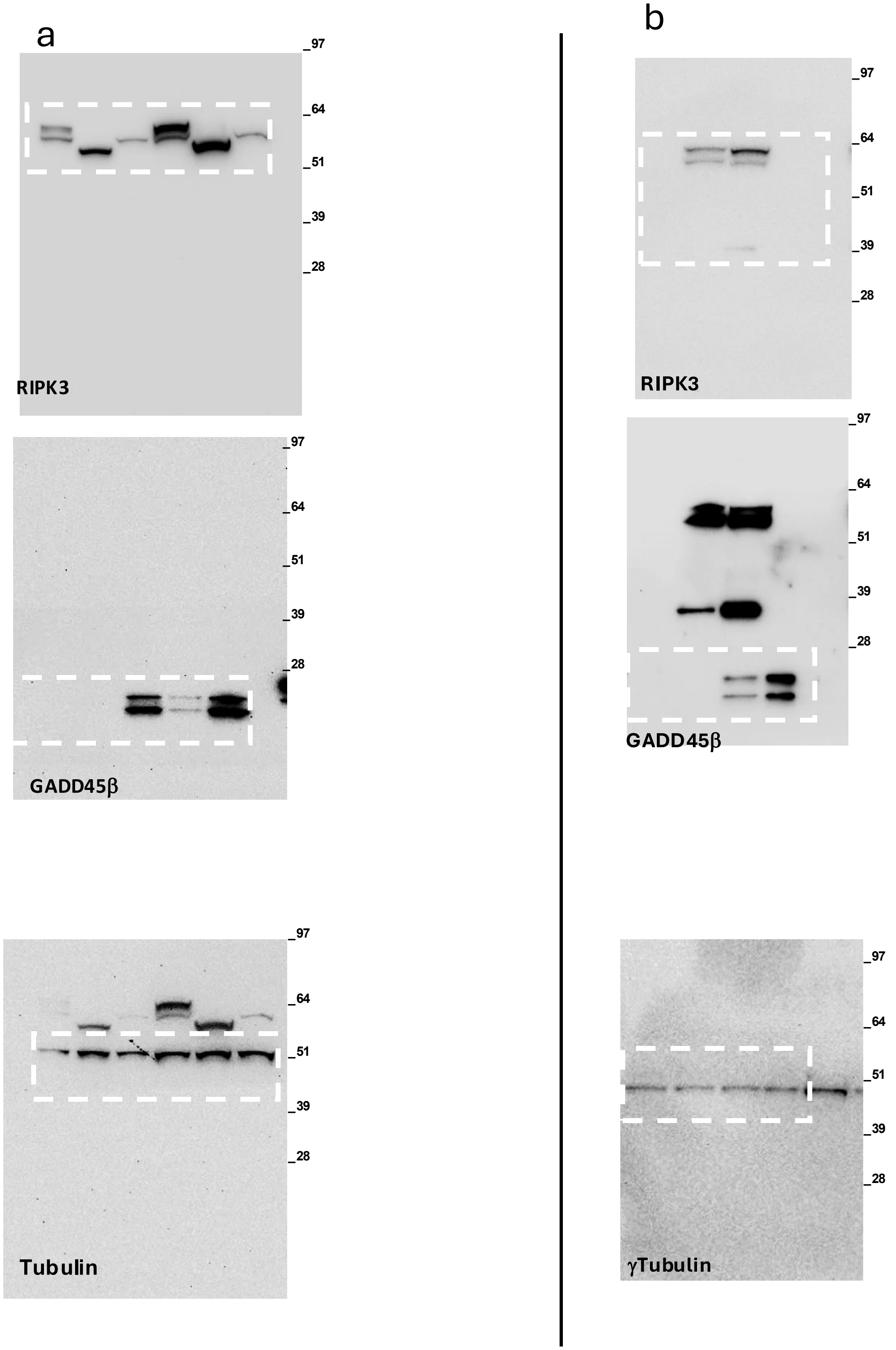
a
b
RIPK3
GADD45β
Tubulin
RIPK3
GADD45β
γTubulin
97
64
51
39
28

Figure S2 a

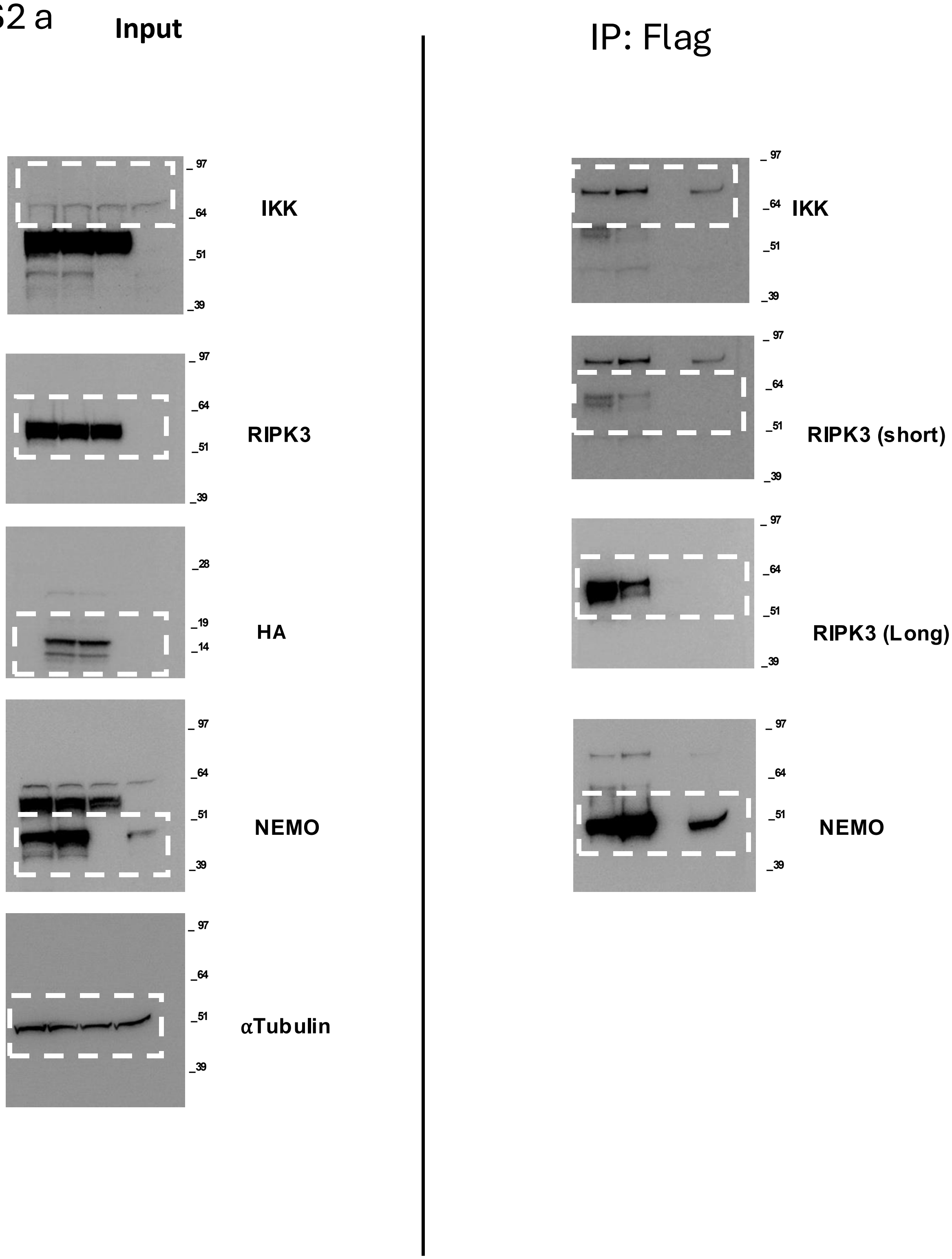
Input
IP: Flag
97
64
51
39
IKK
RIPK3
28
19
14
HA
NEMO
αTubulin
RIPK3 (short)
RIPK3 (Long)

Figure S2 b

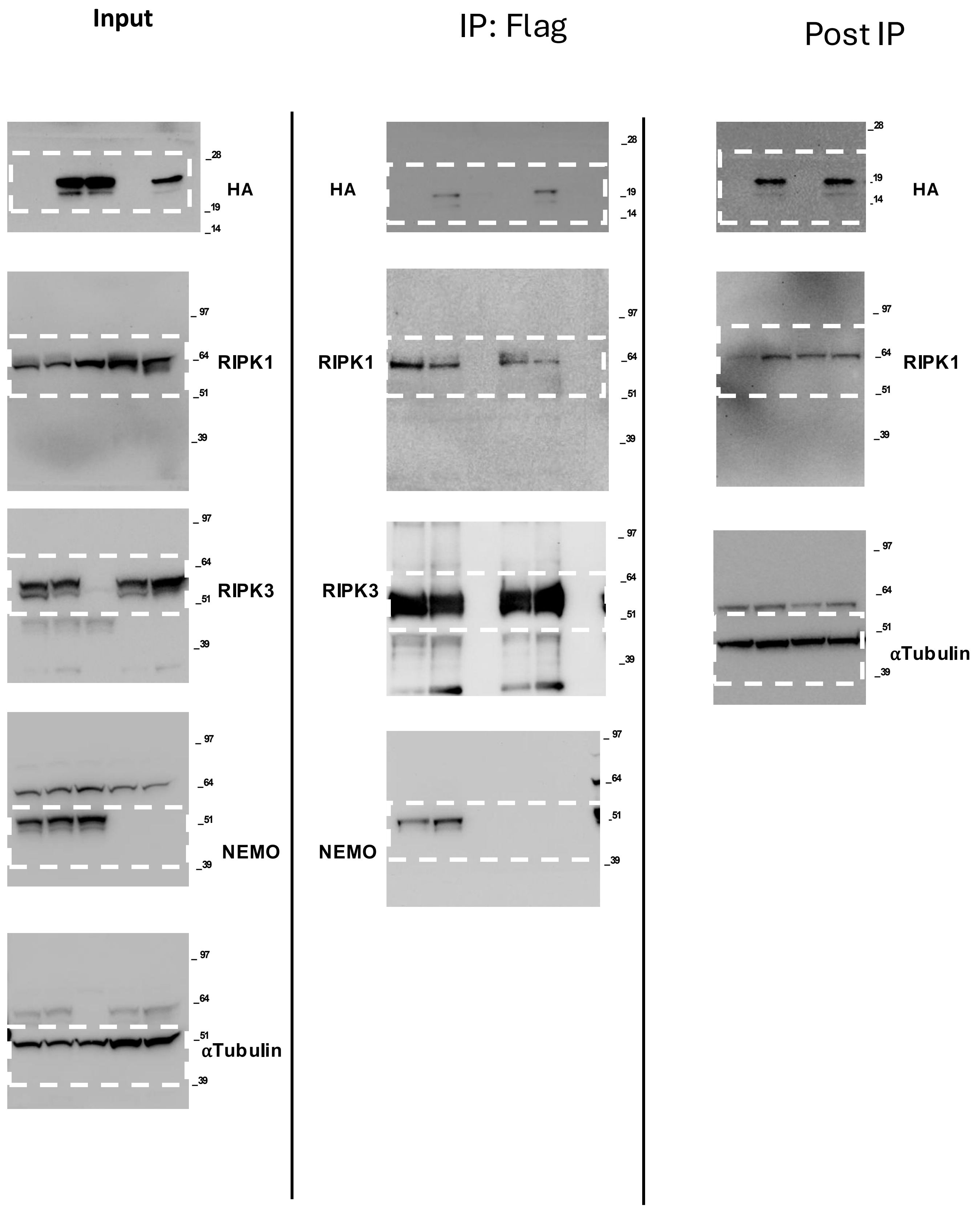
Input
IP: Flag
Post IP
HA
RIPK1
RIPK3
NEMO
αTubulin
28
19
14
97
64
51
39

Figure S2 c

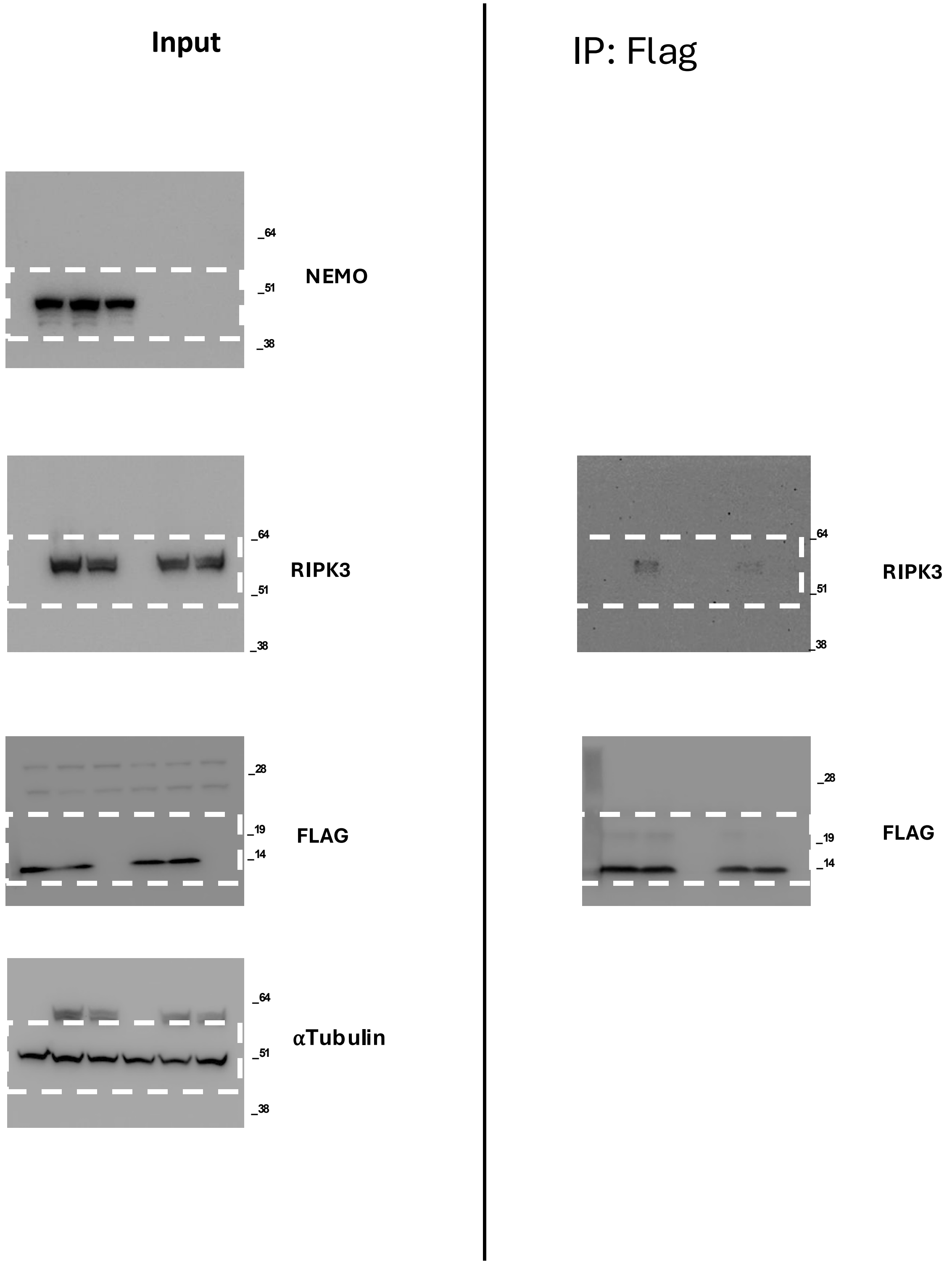

Figure S3

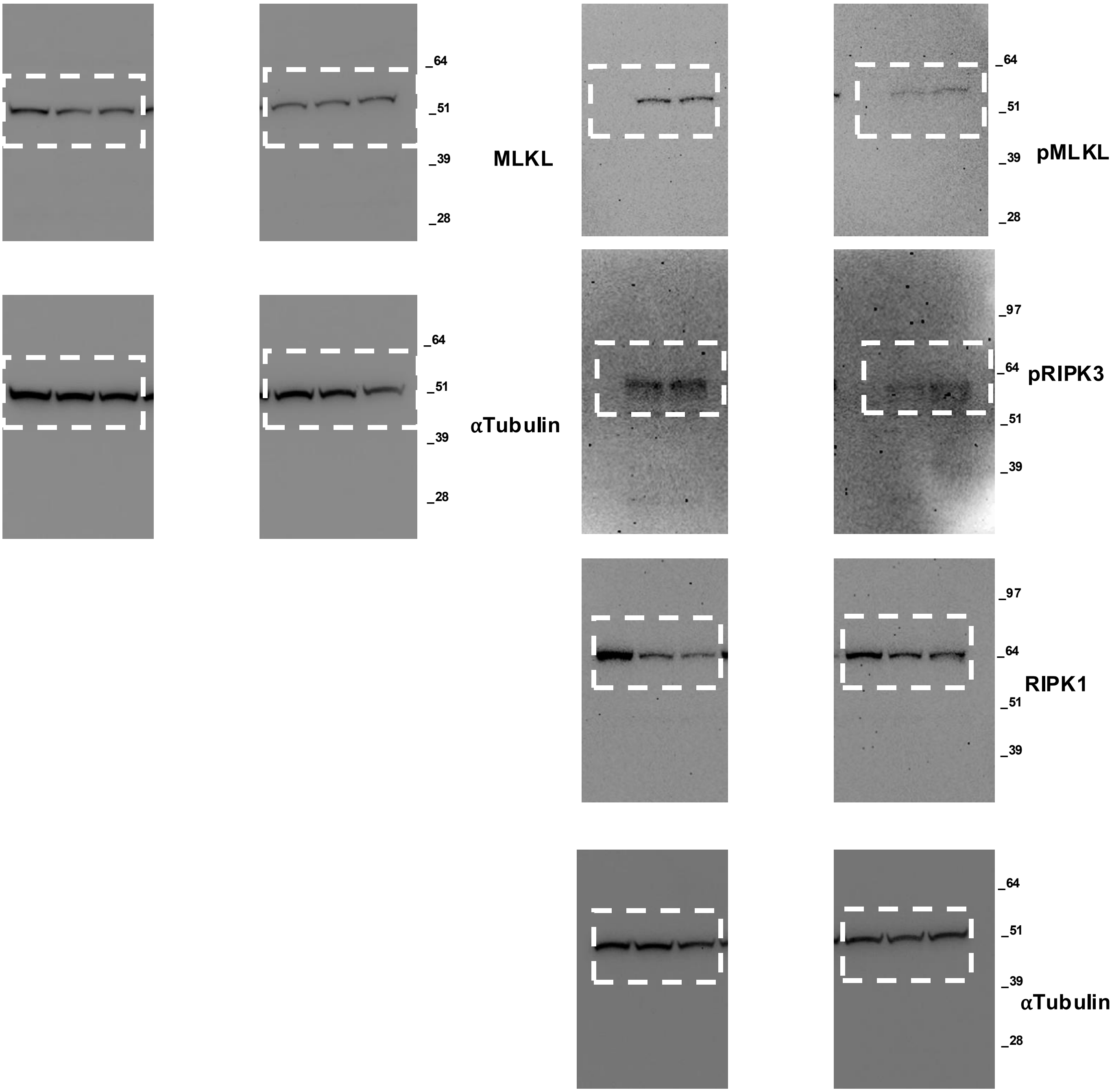

64
51
39
28
MLKL
64
51
39
28
pMLKL
64
51
39
28
αTubulin
97
64
51
39
pRIPK3
97
64
51
39
RIPK1
64
51
39
αTubulin
28
